# Evolutionary history of a vanishing radiation: isolation-dependent persistence and diversification in Pacific Island partulid tree snails

**DOI:** 10.1186/s12862-014-0202-3

**Published:** 2014-09-24

**Authors:** Taehwan Lee, Jingchun Li, Celia KC Churchill, Diarmaid Ó Foighil

**Affiliations:** Museum of Zoology and Department of Ecology and Evolutionary Biology, University of Michigan, 1109 Geddes Avenue, Ann Arbor, MI 48109-1079 USA; Present Address: Marine Science Institute, University of California, Santa Barbara, Santa Barbara, CA 93106-6150 USA

**Keywords:** Oceania, Land snail, Phylogeny, Dispersal, Extinction, Endemism, Conservation

## Abstract

**Background:**

Partulid tree snails are endemic to Pacific high islands and have experienced extraordinary rates of extinction in recent decades. Although they collectively range across a 10,000 km swath of Oceania, half of the family’s total species diversity is endemic to a single Eastern Pacific hot spot archipelago (the Society Islands) and all three partulid genera display highly distinctive distributions. Our goal was to investigate broad scale (range wide) and fine scale (within‐Society Islands) molecular phylogenetic relationships of the two widespread genera, *Partula* and *Samoana*. What can such data tell us regarding the genesis of such divergent generic distribution patterns, and nominal species diversity levels across Oceania?

**Results:**

Museum, captive (zoo) and contemporary field specimens enabled us to genotype 54 of the ~120 recognized species, including many extinct or extirpated taxa, from 14 archipelagoes. The genera *Partula* and *Samoana* are products of very distinct diversification processes. Originating at the western edge of the familial range, the derived genus *Samoana* is a relatively recent arrival in the far eastern archipelagoes (Society, Austral, Marquesas) where it exhibits a stepping‐stone phylogenetic pattern and has proven adept at both intra‐and inter‐ archipelago colonization. The pronounced east–west geographic disjunction exhibited by the genus *Partula* stems from a much older long-distance dispersal event and its high taxonomic diversity in the Society Islands is a product of a long history of within‐archipelago diversification.

**Conclusions:**

The central importance of isolation for partulid lineage persistence and diversification is evident in time-calibrated phylogenetic trees that show that remote archipelagoes least impacted by continental biotas bear the oldest clades and/or the most speciose radiations. In contemporary Oceania, that isolation is being progressively undermined and these tree snails are now directly exposed to introduced continental predators throughout the family’s range. Persistence of partulids in the wild will require proactive exclusion of alien predators in at least some designated refuge islands.

**Electronic supplementary material:**

The online version of this article (doi:10.1186/s12862-014-0202-3) contains supplementary material, which is available to authorized users.

## Background

The Pacific Ocean comprises a third of the Earth’s surface and contains approximately 25,000 oceanic islands [1]. Most are clustered within hot spot and island arc volcanic archipelagoes scattered across Oceania, a tropical region extending from New Guinea to Hawaii and Easter Island [2]. These islands represent highly distinctive evolutionary settings for terrestrial biotas: they have never been connected to continental landmasses and trans-oceanic founder speciation, followed by varying degrees of *in situ* cladogenesis, is the primary diversification process [3-6].

Oceanic island biotas start out with a depauperate and disharmonic composition, *i.e.*, suites of taxa that are prevalent in continental ecosystems, but incapable of dispersing across oceanic barriers, are missing [7]. This facilitates the survival of relict lineages and the evolution of endemic adaptive radiations [3,8,9], resulting in a “diversity and stability paradox” [10]: oceanic island biotas are often species poor, but have high endemism; they appear stable (pending maintenance of biotic isolation), but are susceptible to collapse following anthropogenic disturbance. Indeed, anthropogenic habitat modification/disturbance and human-introduced continental species are the primary drivers of oceanic island extinctions [11]. The small ranges/populations of oceanic island endemics, together with their relative lack of defensive or competitive abilities, render them exceptionally vulnerable to introduced aliens [3,9,12-14].

Although individual islands are often species poor, the cumulative effect of the “diversity and stability paradox” [10] is of global significance: most historical extinctions of birds [15], plants [16] and mollusks [17] worldwide have involved oceanic island endemics. Of particular concern in recent decades has been a major wave of Pacific Island land snail extinctions originating in a misguided biological control program that deliberately introduced a number of alien carnivorous land snails, most notably *Euglandina rosea*, across much of Oceania [18-21]. It is estimated that *E. rosea* alone has probably caused the extinction of 134 Pacific Island land snail endemics [17] and among the hardest impacted are members of the family Partulidae [22-26], the subject of this study.

**Figure 1:**
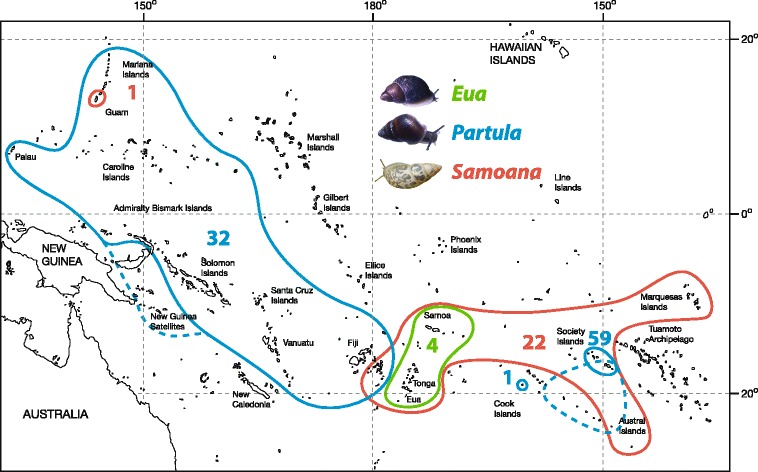
**Partulidae distribution map modified from [** 23 **] to show inferred anthropogenic introductions (dashed lines) of** ***Partula*** **species in Papua New Guinea [** 28 **] and the Cook/Austral Islands [** 34 **].** The number of Society Island *Partula* species is obtained from [26] and [47]. Exemplary snail photographs are by J.B. Burch (*Eua zebrina* and *Partula sinistrorsa*) and J.Y. Meyer (*Samoana attenuata*).

Partulid snails are endemic to the high islands of Oceania *i.e.* islands with sufficient elevation to generate their own precipitation and support rain forest formation. They have attained a spectacular 10,000 km-wide collective range from Palau and the Marianas in the northwest to the Marquesas, Austral and Society Islands in the southeast [23; see Figure 1]. Apart from a single Papua New Guinean record of anthropogenic origin [27,28], they are unknown from adjacent continental habitats, a distribution pattern that strongly implicates biotic factors in setting their distributional limits [3]. Partulid inter-island/archipelago dispersal mechanisms are poorly understood but aerial transport (*via* birds and/or storms) may be more likely than rafting [29-31]. Such chance dispersal events are presumably very rare: most species are endemic to single islands [3,32,33] and the only known partulids with multi-archipelago distributions represent prehistoric anthropogenic introductions [28,34]. Approximately 120 partulid species are currently recognized and half of this nominal diversity is endemic to a single hot spot archipelago, the Society Islands, at the eastern edge of the family’s range (Figure 1). In recent decades, Partulidae have experienced catastrophic extinction, centered on the Society Islands, where most of the archipelago’s 61 endemic species have been extirpated by *Euglandina rosea* [22,23,26,35].

Evolutionary studies of oceanic island lineages share common goals such as the identification of continental sister taxa and the inference of the number, timing and directionality of colonization events among archipelagoes and islands [36]. Unlike Galapagos finches [37] and Hawaiian silverswords [38], for instance, a convincing sister group to Partulidae has not yet been identified [39]; the poorly-studied New Caledonia endemic family Draparnaudiidae (6 known species) is considered the likeliest candidate [40,41]. All available data suggest that Partulidae have had a long evolutionary history in Oceania, dispersing to new islands as older ones sink below sea level. Pilsbry and Cooke [42] partitioned Partulidae into 3 genera (*Partula*, *Samoana* and *Eua*), based primarily on male genital characters, and these have been substantially corroborated by independent molecular character sets [35,39,43-45], with the exception of one Tahitian species complex with highly variable genitalia [46].

*Eua* has the simplest genital anatomy, contains 4 species, and is restricted to the central part of the family’s range: 1 species on ‘Eua (Tonga), 3 in the Samoan Archipelago (Figure 1). *Samoana* contains approximately 23 species and has a disjunct distribution: extensively distributed throughout central and eastern archipelagoes but with a solitary western species, *S. fragilis*, in the Mariana Islands 5000 km from its nearest congener in Fiji (Figure 1). *Partula*, the most speciose genus (~92 species), exhibits a strikingly different disjunct pattern: widespread in western archipelagoes, absent from the central part of the family’s range and, excluding anthropogenic populations [34], restricted to a small subset of eastern islands: 1 species in Rarotonga (Cook Islands), and a remarkable 59 species [26,33,47] in a single hot spot archipelago, the Society Islands (Figure 1).

Kondo and Burch [48] implicitly proposed the following phylogenetic pattern of generic relationships: (*Eua* (*Samoana*, *Partula*)), a topology that has been corroborated by available molecular phylogenies. Land snail gene trees that have included all three genera [39] have placed *Eua* basally, a topology that positions the last common ancestor of extant Partulidae in the central part of the present-day partulid range. Partulid phylogenies rooted with *Eua* species [35,44,45] have consistently recovered reciprocally monophyletic *Samoana* and *Partula* genera. Goodacre and Wade’s [44] nuclear ribosomal phylogeny supported an eastward expansion of *Samoana* species from Samoa to the Society and Marquesan archipelagoes, but the *Partula* clade had a pronounced basal polytomy and yielded little historical insight into this genus’s disjunct distribution.

A comprehensive understanding of partulid diversification processes requires not only a range-wide perspective, but also the development of finer scale within-archipelago and within-island phylogenies. This has been best developed for the Society Island radiation where a number of workers have proposed a “progression rule” model of evolution – lineages sequentially colonize newer islands within an archipelago as they are formed [4] - for the well-studied *Partula* species of Moorea and Tahiti [43,46,49-53]. In this model, neighboring older islands (Leeward group) seeded the younger islands (Windward group), first Moorea, then Tahiti, *via* single colonization events and subsequent speciation occurred *in situ* within each island [43,46,49-53]. This model does not apply to the four endemic Society Island *Samoana* species, two of which have multi-island distributions [35,54,55].

The goal of our study was to build on Goodacre’s pioneering molecular phylogenetic work [44,51,55] by constructing higher-resolution broad scale (range wide) and fine scale (within-Society Island archipelago) partulid phylogenetic relationships for nuclear and mitochondrial genetic markers. Although our geographically- and taxonomically-enhanced sampling scheme was far from exhaustive, and heavily weighted toward Moorean and Tahitian taxa (Table 1), the incorporation of museum and captive (zoo) samples allowed us to genotype numerous extinct and extirpated taxa that span the family’s range; the Solomon Islands being the only major archipelago that lacked a representative. It included all 4 species of *Eua* from 4 islands and 2 archipelagoes, 13 species of *Samoana* from 13 islands and 5 archipelagoes, as well as 37 species of *Partula* sampled from 31 islands and 11 archipelagoes (Table 2). We were particularly interested in investigating the inter-generic relationships and disjunct distributions of *Samoana* and *Partula* [23,33,48]: what is the genesis of such divergent generic distribution patterns, and nominal species diversity levels, in the Western and Eastern segments of the family’s range (Figure 1)?

**Table 1:** **Summary data for partulid taxa genotyped for this study showing the fraction of species in each genus sampled, the number of islands and archipelagoes they were sourced from and the intensive sampling of Tahitian and Moorean** ***Partula*** **populations**

	**Species sampled (species described)**	**Archipelagos represented**	**Islands represented**	**Individuals genotyped**
*Eua*	4 (4)	2	4	6
*Samoana*	14 (25)	5	13	51
*Partula*	37 (99)	11	29	624
Tahitian *Partula*	7 (8)		1	270
Moorean *Partula*	7 (7)		1	140
Total	55 (128)	14	41	681

**Table 2:** **Taxonomic designations, sampling locations, IUCN Red List Status and sources for the 54 partulid species genotyped in this study**

**Taxonomy**	**Island, Archipelago**	**IUCN Red list status**	**Source/Date sampled**
*Eua globosa* Pilsbry & Cooke, 1934	′Eua, Tonga	Critically Endangered	UMMZ/1970
*E. expansa* (Pease, 1872)	Savai‘i, Samoa	Unevaluated	FMNH/1965
*E. montana* (Cooke & Crampton, 1930)	Upolu, Samoa,	Unevaluated	FMNH/1965
*E. zebrina* (Gould, 1847)	Tutuila, Samoa (Am.)	Endangered	UMMZ/1970
*Samoana fragilis* (Ferussac, 1821)	Guam, Marianas	Critically Endangered	FMNH/1945
*S. abbreviata* (Mousson, 1869)	Tutuila, Am. Samoa	Critically Endangered	UMMZ/1970
*S. conica* (Gould, 1847)	Tutuila, Am. Samoa	Unevaluated	UMMZ/1970
*S. thurstoni* (Cooke & Crampton, 1930)	Ofu, Am. Samoa	Endangered	FMNH/1975
*S. canalis* (Mousson, 1865)	Savai‘i, Samoa	Endangered	FMNH/1965
*S. stevensoniana* (Pilsbry, 1909)	Savai‘i, Samoa	Unevaluated	FMNH/1965
*S. margaritae* (Crampton & Cooke, 1953)	Rapa, Australs	Vulnerable	Fontaine/2002
*S. oreas* (Crampton & Cooke, 1953)	Raivavae, Australs	Critically Endangered	Fontaine/2002
*S. attenuata* (Pease, 1864)	Raiatea/Moorea/Tahiti, Society	Critically Endangered	UMMZ/1970 Coote/2005
			Meyer/2006
			Hickman/2006
*S. burchi* Kondo, 1973	Tahiti, Society	Critically Endangered	UMMZ/1970
			Coote/2005
*S. diaphana* Crampton & Cooke, 1953	Moorea/Tahiti, Society	Endangered	UMMZ/1970
			Coote/2006, 2007
			Holland/2004
*S. bellula* (Hartman, 1885)	Ua Pou, Marquesas	Critically Endangered	Holland/2005
*S. decussatula* (Pfeiffer, 1850)	Tahuata/Hiva Oa, Marquesas	Critically Endangered	ZSL/1995
			Holland/2004
			Coote/2005
*S. strigata* (Pease, 1868)	Nuku Hiva, Marquesas	Critically Endangered	Holland/2004
*Partula calypso* Semper, 1865	Babeldaob, Palau	Critically Endangered	FMNH/2005
*P. thetis* Semper, 1865	Ulong /Babeldaob/Ngeruktabel, Palau	Endangered	FMNH/1995, 1998, 2006
*P. gibba* Ferussac, 1821	Guam/Saipan, Marianas	Critically Endangered	FMNH/1958
			UMMZ/1970
			ZSL/1970
*P. radiolata* Pfeiffer, 1846	Guam, Marianas	Critically Endangered	FMNH/1995
			ZSL/1995
*P. emersoni* Pilsbry, 1913	Pohnpei, Carolines	Critically Endangered	Holland/2011
*P. carteriensis* (Quoy & Gaimard, 1832)	New Britain, Bismarcks	Data Deficient	FLMNH/2005
*P. similaris* Hartman, 1886	Woodlark/Boiaboiawaga/Goodenough, d’Entrecasteaux	Data Deficient	UMMZ/1966
			FMNH/2002
			FLMNH/2003
*P. auraniana* Hartman, 1888	Toga/Tegua/Loh/Metoma/Hiu,Torres, Vanuatu	Endangered	Fontaine/2009
*P. turneri* Pfeiffer, 1860	Erromanga/Tanna, Vanuatu	Unevaluated	FMNH/1972
			FLMNH/1984
*P. lirata* Mousson, 1865	Thikombia-i-lau, Fiji	Unevaluated	ZSL/1999
*P. subgonochila* Mousson, 1871	Alofi, Wallis & Futuna	Critically Endangered	ZSL/2007
*P. assimilis* Pease, 1868	Rarotonga, Cooks	Unevaluated	Coote/2004
*P. lutea* Lesson, 1831	Bora Bora, Society	Extinct	UMMZ/1970
*P. dentifera* Pfeiffer, 1853	Raiatea, Society	Extinct in the Wild	UMMZ/1970
			ZSL/1991
*P. faba* (Gmelin, 1791)	Raiatea, Society	Extinct in the Wild	UMMZ/1970
			ZSL/1991-2
*P. hebe* (Pfeiffer, 1846)	Raiatea, Society	Extinct in the Wild	UMMZ/1970
			ZSL/1991
*P. labrusca* Crampton & Cooke, 1953	Raiatea, Society	Extinct	ZSL/1992
*P. meyeri* Burch, 2007	Raiatea, Society	Critically Endangered	Meyer/2006
*P. tristis* Crampton & Cooke, 1953	Raiatea, Society	Extinct in the Wild	ZSL/1991
*P. turgida* (Pease, 1865)	Raiatea, Society	Extinct	ZSL/1991
*P. arguta* (Pease, 1865)	Huahine, Society	Extinct	ZSL/1991
*P. rosea* Broderip, 1832	Huahine, Society	Extinct in the Wild	ZSL/1987
*P. varia* Broderip, 1832	Huahine, Society	Extinct in the Wild	ZSL/1991, 1994
*P. aurantia* Crampton, 1932	Moorea, Society	Extinct	UMMZ/1970
*P. exigua* Crampton, 1917	Moorea, Society	Extinct	UMMZ/1970
*P. mirabilis* Crampton, 1924	Moorea, Society	Extinct in the Wild	UMMZ/1970
			ZSL/1984-5
*P. mooreana* Hartman, 1880	Moorea, Society	Extinct in the Wild	UMMZ/1970
			ZSL/1982, 1985
*P. suturalis* Pfeiffer, 1855	Moorea, Society	Extinct in the Wild	UMMZ/1970
			ZSL/1980-6
*P. taeniata* (Mörch, 1850)	Moorea, Society	Critically Endangered	UMMZ/1970
			ZSL/1981-6
			Coote/2005-7
			Hickman/2006
			Meyer/2006
*P. tohiveana* Crampton, 1924	Moorea, Society	Extinct in the Wild	ZSL/1982
*P. affinis* Pease, 1867	Tahiti, Society	Critically Endangered	UMMZ/1970
			ZSL/1995
*P. clara* Pease, 1864	Tahiti, Society	Critically Endangered	UMMZ/1970
			ZSL/1995, 1997
			Coote/2004-7
*P. filosa* Pfeiffer, 1853	Tahiti, Society	Extinct	UMMZ/1970
*P. hyalina* Broderip, 1832	Tahiti, Society	Vulnerable	UMMZ/1970
	Mangaia/ Mauke, Cooks		ZSL/1996
	Rimatara/Rurutu/Tubuai/Raiavavae, Australs		Coote/2004-7
			Fontaine & Gargominy/2002-4
			McCormack/2006
*P. nodosa* Pfeiffer, 1851	Tahiti, Society	Extinct in the Wild	UMMZ/1970 ZSL/1984
*P. otaheitana* (Brugière, 1789)	Tahiti, Society	Critically Endangered	UMMZ/1970
			ZSL/1995
			Coote/2005-7
*P. producta* Pease, 1865	Tahiti, Society	Extinct	UMMZ/1970

See Additional file 3: Table S1 for detailed information, including museum voucher and GenBank numbers, on the genotyped snails.

## Results & discussion

### Intergeneric relationships

**Figure 2:**
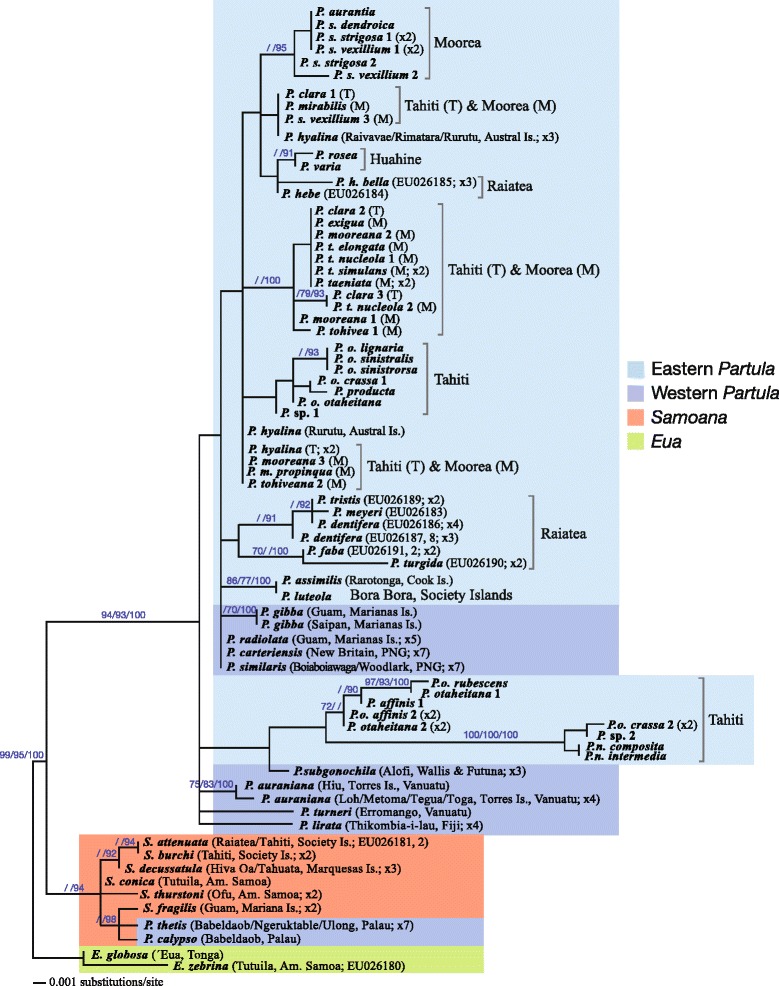
**Large nuclear ribosomal (28S) rDNA Maximum Likelihood phylogenetic tree of Partulidae.** The two *Eua* species were the designated outgroups. Support valves ≥50 are shown above the pertinent nodes respectively for Maximum Parsimony (left) and Maximum Likelihood (middle) bootstrap valves as well as Bayesian posterior probability values (right).

**Figure 3:**
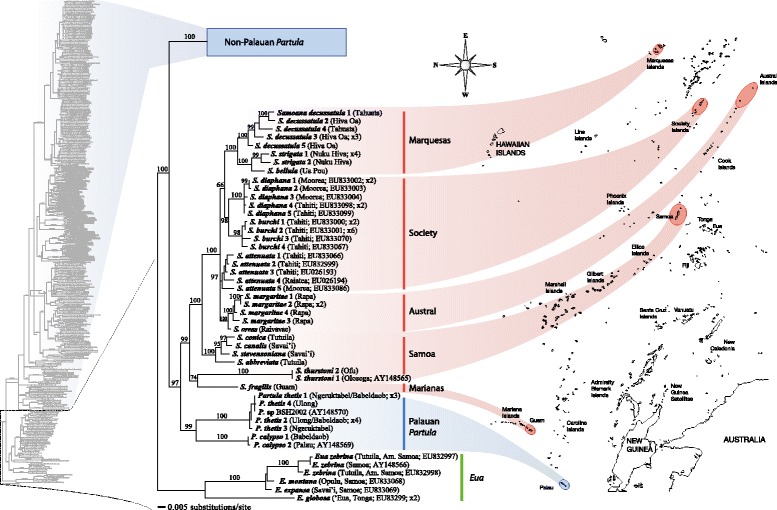
**Bayesian phylogenetic tree of the Partulidae mt COI dataset.** The entire tree is shown in profile together with an expanded detail of the basal segment composed of the designated outgroup genus *Eua*, members of the genus *Samoana* (with red background highlighting) and two Palauan *Partula* species. Posterior probability values ≥50 are given on their respective nodes.

Previous partulid molecular phylogenies [35,39,43-45] have either ignored or under-sampled Western Pacific taxa, the most comprehensive representation being 4 western species of *Partula* [44]. We were able to incorporate 14 Western Pacific species (Table 2) in our analyses, including for the first time *Samoana fragilis,* the sole western member of that genus, and 2 of the 3 endemic *Partula* species in Palau, the westernmost archipelago of the family’s range (Figure 1). Twelve of the Western Pacific partulids clustered as expected with Central/Eastern Pacific congeners in our molecular phylogenetic trees. However, the two Palauan species, *P. thetis* and *P. calypso*, were topologically distinct from all of their congeners, forming discrete and robustly supported clades with *Samoana* species. For our relatively conserved marker, the large nuclear ribosomal gene fragment, the two Palauan *Partula* species formed a shallow polytomy with *Samoana fragilis*, within a polytomous *Samoana* clade (Figure 2). The faster-evolving mt COI marker yielded a more fully resolved inference of Palauan partulid genealogical relationships – see Figure 3 for the salient segment of this much larger phylogenetic tree (the complete topology is available in Additional file 1: Figure S1). Here, *P. thetis* and *P. calypso* were sister to all genotyped *Samoana* species.

The unexpected phylogenetic placement of the two Palauan *Partula* taxa with *Samoana* species raises a question concerning their generic status. Pilsbry and Cooke [42] distinguished the genera *Partula* and *Samoana* primarily on male genital characters: the latter being differentiated into distinct epiphallus and penis segments connected by an extension of the penial retractor muscle that is absent in other partulids. Kondo’s Ph.D. thesis [56] contains detailed diagrams of the reproductive tracts of the three endemic Palauan species (*P. thetis*, *P. calypso* and *P. leucothoe*, the latter was unavailable for this study) and all three unambiguously displayed the male genital anatomy characteristic of the genus *Partula*. These anatomical data, together with the robust, well-resolved mt gene tree topology (Figure 3) demonstrate that the genus *Partula* is paraphyletic and that the distinct genital anatomy of *Samoana* species is derived. In contrast to Kondo and Burch’s [48] hypothesis of intergeneric relationships - (*Eua* (*Samoana*, *Partula*)) - our data are consistent with (*Eua* (*Partula1* (*Partula2, Samoana*))) where *Partula1* represents all non-Paluan members of the genus and *Partula2* represents the two genotyped Palauan species.

### West–east dispersal of *Samoana*

The most remarkable feature of the distribution of *Samoana* is the presence of a single isolated species, *S. fragilis*, bearing the characteristic *Samoana* genital anatomy [56], in the Western Pacific (Figure 1). Genotyping *S. fragilis* museum specimens sampled in 1945 allowed us to put this geographic disjunction into a phylogenetic context for the first time. Our two molecular markers yielded very different levels of phylogenetic resolution. The more conserved nuclear large ribosomal gene fragment produced a shallow clade with a basal polytomy such that interrelationships among Western (including the two Palauan *Partula* species) and Eastern *Samoana* taxa were unresolved (Figure 2). The mt marker provided much better resolution (Figure 3) and we base our discussion of the evolutionary history of *Samoana* on this topology. It shows that the *Samoana* clade is anchored in the West by its exclusive sister relationship with Palauan *Partula* species and by the placement of the Marianas species *S. fragilis* at its base - the sole western species is not a derived founder lineage (Figure 3). This is a somewhat surprising result because it places the inferred evolutionary origins of the genus not in the Eastern Pacific, home to 21/22 *Samoana* species (Figure 1), but instead in the far western portion of the partulid range (Figure 3).

The mt gene tree topology (Figure 3) is consistent with a western origin of the genus *Samoana* followed by progressive eastward colonization of derived lineages. *S. fragilis* is sister to the Samoan congener *S. thurstoni*, although the posterior probability value for this node is low. A derived clade, that approximates a stepping-stone dispersal pattern from West to East, is evident for the easternmost archipelagoes with Samoa as the regional source. Three closely-related Samoan species – *S. conica*, *S. stevensonia* and *S. abbreviata* – are robustly sister to derived congers in the Society, Austral and Marquesan archipelagoes, the latter two forming monophyletic clades. Reconstructing the multi-archipelagic dispersal pathways of these Eastern lineages is complicated by a basal polytomy involving Austral taxa and two Society Island lineages (Figure 3). However, the sister status of the Marquesan taxa with two Society Island congeners, *S. diaphana* and *S. burchi*, is consistent with an earlier inference [44] of dispersal and colonization among these two archipelagoes. Our results corroborate a previous allozyme study [57] regarding Marquesan monophyly and adaptive radiation – Marquesan taxa exhibit a diversity of distinct shell phenotypes. Interestingly, so do the two Society island montane forest sister taxa: the thin-shelled *S. diaphana* (Moorea and Thaiti) and the thick-shelled Tahitian endemic *S. burchi* [54].

Although our data provide new insights into the evolution and biogeography of the genus *Samoana*, they raise obvious follow-on questions: if the genus originated in the West, why does almost all the extant diversity occur in Eastern archipelagoes and why are *Samoana* species missing from a huge swathe of the family’s range from the Marianas to Fiji (Figure 1)? We consider it unlikely that the genus extended its range across this ~5000 km gap in a single dispersal event and hypothesize that at least some intermediate archipelagoes such as the Caroline Islands, Bismarck Archipelago, Solomon Islands, Santa Cruz Islands and Vanuatu may have supported populations at some time in the past. Present day absence of the genus *Samoana* from at least some of these archipelagoes is probably not due to a lack of dispersal ability. Our data indicate that a Samoan lineage was capable of stepping-stone colonization of Eastern archipelagoes up to 3000 km distant, a range that ostensibly puts the Western archipelagoes of Vanuatu, Santa Cruz and the Solomon Islands within equivalent reach. The absence of the genus *Samoana* from much of the Western range of the family may stem from (as yet unidentified) ecological factors that have driven original populations to extinction and prevented rare long-distant migrants from reestablishing new populations.

### West–east *Partula* disjunction – product of a single dispersal event

The ~2000 km geographic disjunction – from the Lau Islands of Fiji to Rarotonga in the Cook Islands – separating Western and Eastern members of the genus *Partula* (Figure 1) has yet to be put into a comprehensive phylogenetic framework. Although our sampling of the genus *Partula* is heavily weighted toward Eastern populations, we have genotyped 11 species from across the Western range (Marianas, Palau, Carolines, Bismarck Archipelago and the Massim Region of Papua New Guinea, Vanuatu, Wallis and Futuna, Fiji). This broad sampling allows some confidence that we have captured much of the regional phylogenetic framework, if not all of its detail. Similarly, for the Eastern *Partula* species, our taxon sampling is geographically comprehensive: the 26 species genotyped include the sole non-anthropogenic Cook Island species, *P. assimilis*, as well as representatives from the all of the salient Society Islands except for Tahaa, which shares the same lagoon as Raiatea.

**Figure 4:**
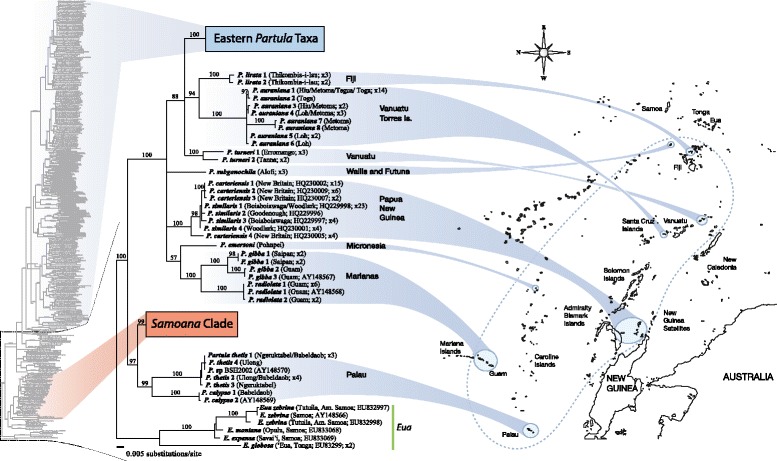
**Bayesian phylogenetic tree of the Partulidae mt COI dataset.** The entire tree is shown in profile together with an expanded detail of the phylogenetic relationships of the Western Pacific species of *Partula* species. The genus *Eua* is the designated outgroup and see Figure 3 for details of the collapsed *Samoana* clade. Posterior probability values ≥50 are given on their respective nodes.

Our phylogenetic analyses of the large nuclear gene fragment consistently produced a very poorly resolved topology for the non-Palauan *Partula* species, as previously reported [44], *e.g.*, Figure 2 contains two polytomies, each with a mix of Western and Eastern species. We are therefore basing our inferences of West–east *Partula* relationships on the mt topology. As shown in Figure 4, the Western taxa place at the base of the non-Palauan *Partula* clade separated by a robust (PPS = 100) node from all Eastern congeners. This is consistent with a single dispersal event from West to East (Figure 1).

Phylogenetic relationships among the non-Palauan Western *Partula* taxa were obscured by a basal polytomy (Figure 4). Within-archipelago monophyly was evident only for Marianas taxa and these were weakly sister to the Micronesian *P. emersoni*. The phylogenetic affinities of Papua New Guinean populations of *P. carteriensis* and *P. similaris*, flagged as likely anthropogenic introductions [28], remained uncertain. The two easternmost taxa had distinct topological placements: *P. subgonochila* (Wallis & Futuna) was a member of the basal polytomy whereas the Fijian *P. lirata* was sister to one of the Vanuatu species, *P. auraniana* (Torres Islands). These disparate results imply that a more comprehensive sampling of Western taxa is required to capture a high-resolution understanding of regional evolutionary history. Unfortunately, this will have to be accomplished quickly given the ongoing decline and extirpation of many Western Pacific partulid populations and species [24,58-61].

### Eastern *Partula* diversification

**Figure 5:**
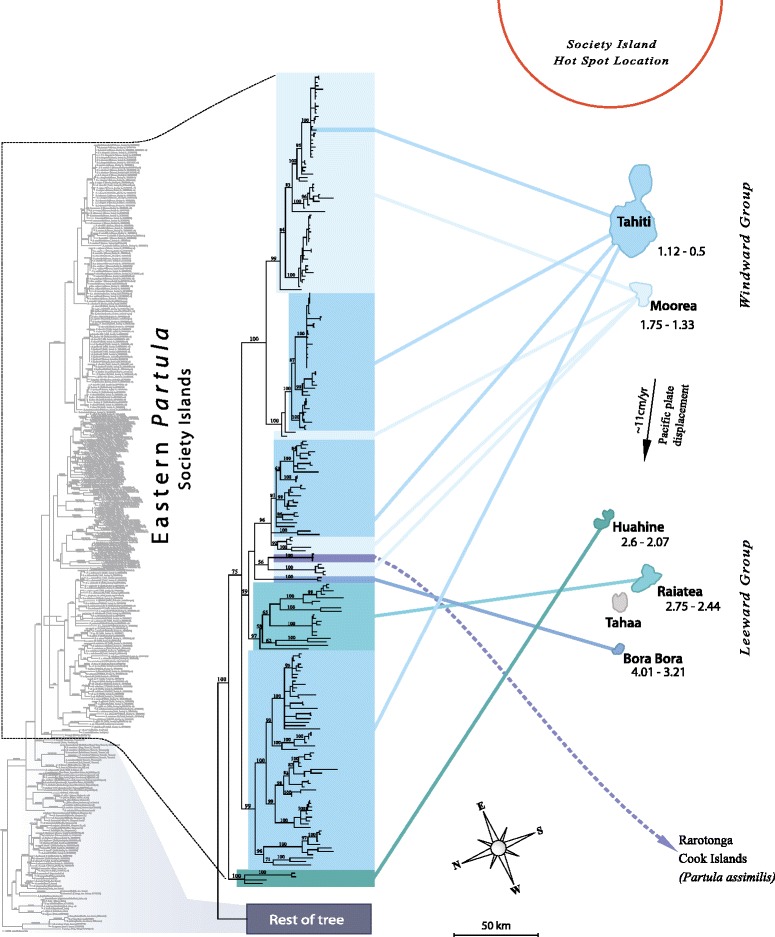
**Bayesian phylogenetic tree of the Partulidae mt COI dataset.** The entire tree is shown in profile together with an expanded view of the phylogenetic relationships of the Eastern Pacific species of *Partula*. Due to space restrictions, Eastern *Partula* species are identified here by color-coded source island only – see Additional file 1: Figure S1 for a full-scale topology containing taxonomic details and GenBank numbers. See Figures 3 & 4 for details of the non-magnified basal portion of the tree, including the outgroup genus *Eua*, the genus *Samoana* and Western Pacific *Partula* species. Posterior probability values ≥50 are given on their respective nodes. Respective geological age range estimates in Mya are available below each island and are sourced from [81] for Bora Bora and from [82] for Tahiti, Moorea, Huahine and Raiatea. The approximate positioning of the Society Island hotspot is from [67].

Figure 5 shows, in outline form, the mt tree topology of the Eastern *Partula* clade labeled by source island – a full-sized topology containing details of individual genotype identification is available in Additional file 1: Figure S1. Apart from major gaps for the islands Tahaa (0/6 species) and Raiatea (7/34 species), sampling of the Society Islands endemic *Partula* radiation was almost complete: Bora Bora (1/1), Huahine (3/3), Moorea (7/7) and Tahiti (7/8 – the missing species, *P. cythera*, has not been seen since its discovery on a remote interior mountain slope in the 1920’s [62]).

The only Eastern *Partula* species not endemic to the Society Islands – *P. assimilis* of Rarotonga (Cook Islands) – is positioned within the Society Island clade (Figure 5), weakly sister to a minor, divergent Moorean *P. suturalis* clade and to the Bora Bora endemic *P. lutea*, with which it shares a large nuclear ribosomal genotype (Figure 2) as well as allozymic affinities [43]. We agree with Johnson *et al*. [43] that *P. assimilis* probably represents a founder lineage from a Society Islands source; the only know *Partula* transplant from that hotspot archipelago apart from anthropogenic populations of the Tahitian endemic *P. hyalina* [34].

Concerning the Society Islands topology, a number of important stem nodes within the clade were poorly supported and, consistent with parallel studies on this archipelago’s biota [53], there was little agreement with progression rule [4] expectations for the archipelago, *i.e.*, topological congruence with the chronological sequence of island formation. Only one (Huahine) of three older Society Islands represented in the topology (Bora Bora, Raiatea, Huahine; all members of the Leeward Island subgroup) positioned basally. The oldest Society Islands with partulids, Bora Bora and Raiatea, placed in derived positions, weakly sister to a heterogeneous assemblage that included the Rarotonga species, two Moorean clades and one Tahitian clade (Figure 5).

One consistent topological difference between the Leeward and Windward Island lineages concerned their degree of within-island monophyly. Genotyped snails from each Leeward Island formed well-supported (PPS ≥97) single island-specific clades. Snails from each Windward Island formed four discrete lineages, three of which had exclusive phylogenetic relationships (PPS ≥96) with snails in the other Windward island, as described in detail in an earlier study [35]. However, the phylogenetic affinities of the largest and most deeply branched of the four Tahitian mt clades remained enigmatic. This robust (PPS = 99) Tahitian clade positioned on a basal polytomy that encompassed both Leeward and Windward Island lineages and it lacked an identifiable sister lineage, either on Moorea, or on any of the other Society Islands (Figure 5).

The Figure 5 topology is incompatible with the prevailing speciation model for Moorean and Tahitian *Partula* that views all congeners on each Windward Island as the product of a single colonization event: a Leeward Island source for Moorea and a single Moorean source for the subsequent colonization of Tahiti [49]. The model has a narrow base of empirical support: allozyme phylogenies from 24 species of *Partula* that have been weighted on the basis of allele frequency [43,49]. These data overturned previous inferences – based on reproductive relationships and morphological similarities – that Tahiti was colonized by multiple Moorean lineages [63]. However, the allozyme data were less than robust: an unweighted (UPGMA) analysis of the same allozyme dataset yielded a substantially distinct topology in which the Tahitian species were no longer monophyletic [43] and a later reanalysis yielded another topology consistent with back-migration from Tahiti to the southern part of Moorea [64]. Salient DNA phylogenies, whether using nuclear ribosomal [44, this study] or mt [35, 50–52, this study] markers have failed to recover the model’s predicted topology. The mt studies, in particular, have consistently revealed the presence of multiple exclusive sister relationships among subsections of Moorean and Tahitian *Partula* mt treespace [35, 50–52, this study].

Despite these incongruences, the Windward Island *Partula* speciation model [49] has remained the consensus conceptual framework for speciation studies of Moorean and Tahitian *Partula* species: speciation is viewed as having occurred *in situ* within each island [43,46,49-53,65]. Consistent with this view, examples of Moorean/Tahitian mt polyphyly have been interpreted either as being due to convergent molecular evolution (for restriction fragment length polymorphism data [23,50], or to retained ancestral mt polymorphisms [51-53], rather than being products of inter-island gene flow involving multiple discrete lineages. However, both of these non-gene flow interpretations are problematic. The large majority of mt COI nucleotide substitutions observed among Moorean and Tahitian taxa are synonymous (data not shown) and are therefore unlikely to stem from selection-driven convergent evolution. The ancestral polymorphism interpretation requires a vicariant model of inter-island genetic differentiation that is inapplicable to Moorea and Tahiti, two spatially discrete islands that have never been joined [66,67].

We propose a new speciation model for Tahitian *Partula* taxa that involves four discrete founding lineages. Three of the four have explicit phylogenetic ties to Moorean congeners, as detailed earlier [35], but the largest, comprising half of Tahitian mt treespace, is of undetermined provenance (Figure 5). This new model is consistent with parallel Society Island studies that have uncovered a role for multiple independent colonizations in the evolution of Tahiti’s endemic biota [53].

### Age of the Society Islands *Partula* radiation

A standout feature of the biogeography of Partulidae is the markedly asymmetric distribution of alpha diversity across the family’s 10,000 km range (Figure 1). Half of the nominal species diversity is endemic to six Society Islands spanning a mere 320 km of Oceania with a quarter being endemic to a single island: the 167 km^2^ Raiatea [23]. The extraordinary concentration of 59 species of *Partula* within this single hotspot archipelago raises questions of taxonomic equivalency (species descriptions are typically based on shell phenotype distinctions) and lineage persistence across the range. While we cannot engage meaningfully here with the fraught topic of *Partula* species designations [52,64,68], we can address the issue of lineage persistence by constructing a time-calibrated phylogeny for our molecular dataset.

**Figure 6:**
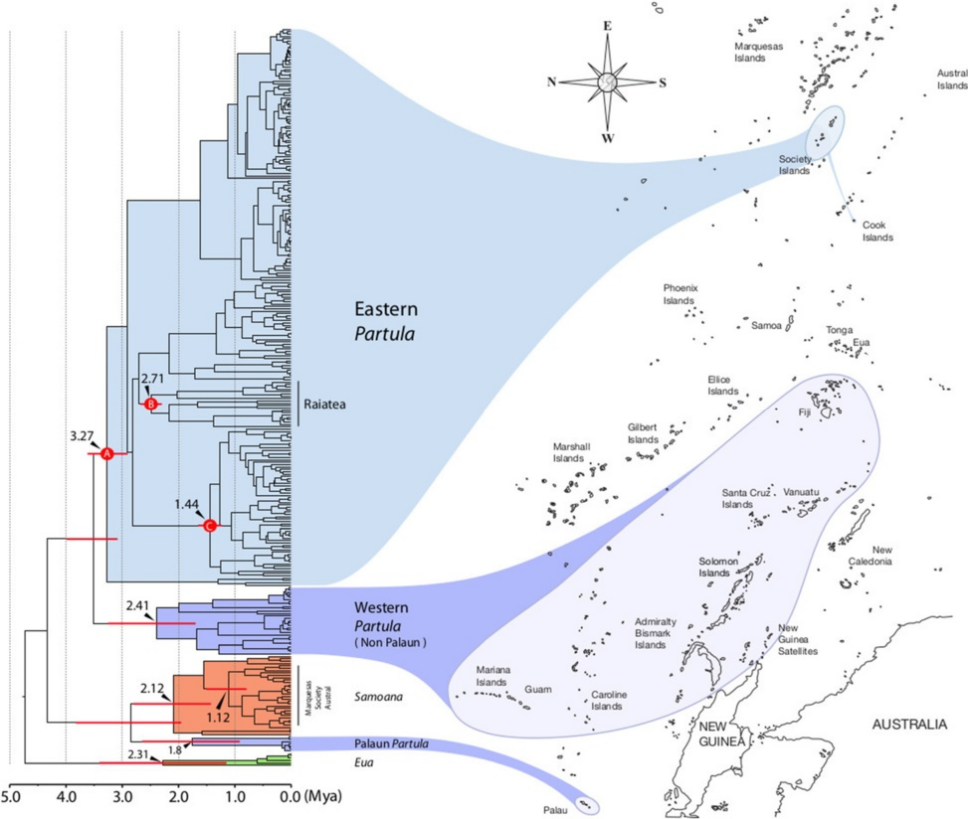
**A time calibrated mt COI BEAST phylogeny of Partulidae.** Red dots represent calibration points based on Society Island ages. A: Bora Bora, 3.27 Mya [81], on the node supporting the Eastern *Partula* clade; B: Raiatea, 2.71 Mya [82], on the node supporting the Raiatean *Partula* clade; C: Tahiti, 1.44 Mya [82], on the node supporting the oldest exclusively Tahitian *Partula* clade. Also given on their respective nodes are the estimated divergence times of the non-Palauan Western *Partula* clade (2.41 Mya), the genus *Samoana* (2.12 Mya), the Eastern *Samoana* clade (Marquesas, Society and Austral archipelagoes; 1.12 Mya), Palauan *Partula* (1.8 Mya) and the genus *Eua* (2.31 Mya). 95% Highest Posterior Density (HPD) age intervals of major clades are shown as red bars. See Additional file 2: Figure S2 for a full-scale version of this topology containing taxonomic details and date estimates for each node.

Figure 6 shows, in outline form, the time-calibrated Partulidae mt tree obtained with BEAST (see details of the topology in Additional file 2: Figure S2). Three calibration points based on the geological ages of Bora Bora, Raiatea and Tahiti, were used to date nodes supporting the entire Society Island clade, the endemic Raiatean clade and the largest endemic Tahitian clade, respectively. The BEAST topology differed little from that of the Bayesian analyses (Figures 3,4 and 5) except that the non-Palauan Western *Partula* species now formed a discrete clade, sister to their Eastern congeners (Figure 6). It yielded a greater inferred age for the Society Island *Partula* radiation, both for the entire archipelago (3.27 Mya), and for the single island of Raiatea (2.71 Mya), than for the much more widespread sister Western *Partula* clade (2.41 Mya), the genus *Eua* (2.31 Mya) and the family range-spanning genus *Samoana* (2.12 Mya), although note that the 95% Highest Posterior Density age intervals for many of these nodes overlap (Figure 6).

These time-calibrated data imply that the heightened alpha diversity characteristic of the Society Island *Partula* radiation, and of the island of Raiatea, resulted at least in part from a longer uninterrupted diversification timeframe than was available elsewhere in the family’s range. For this within-archipelago radiation, cladogenesis has apparently unfolded in a qualitatively different manner to that experienced by partulids elsewhere, being characterized (until very recently) by greater lineage longevity coupled with much less lineage dispersal. Within the archipelago, the hyper-speciose Raiatean radiation is correlated with that island’s age, area and relief. Despite its relatively advanced age (Figure 5), Raiatea is second only to Tahiti in area and has retained the high island profile (1017 m maximum elevation) required for autonomous rainfall generation and rain forest formation [69,70]. Outside of the Society Island *Partula* radiation, the small Palauan *Partula* and *Eua* clades also show evidence for long-term persistence within modest ranges, but lineage diversification in the Western *Partula* and *Samoana* crown clades occurred across much more extensive ranges and necessarily involved multiple episodes of among-archipelago dispersal (Figures 3 and 6).

### Divergent histories of Eastern *Partula* and *Samoana* radiations

Although the genera *Partula* and *Samoana* overlap in the eastern edge of the family’s range, co-occurring in the Society Islands, they appear to have experienced distinct patterns of regional cladogenesis [35]. *Partula* has many more regional species (59 vs. 15 respectively), but *Samoana* has a greater eastern range, encompassing the Marquesas and Austral Archipelagoes as well as the Society Islands (Figure 1). They also differ in their regional phylogenetic/population genetic profiles: eastern *Samoana* species have much lower collective genetic diversity levels and more pronounced phylogenetic cohesiveness [35,55,57]. The BEAST topology (Figure 6) yielded new insights into the evolutionary origins of these regional distinctions, showing that the genus *Partula* established eastern populations much earlier (3.27 Mya) than did *Samoana* (1.12 Mya). This divergent chronology probably contributed to the regional generic species richness disparity, but not to their distributional disparity. Despite its much longer regional timeframe, the genus *Partula* has effectively been marooned on the Society Islands, establishing just one inter-archipelago founder species in Rarotonga (Cook Islands), excepting anthropogenic introductions of *P. hyalina* [34]. In contrast, the later arriving Eastern *Samoana* lineage has proven to be a much more effective regional disperser, on both intra-archipelago (many species have multi-island distributions: 33, 35, 54, 55, 57] and inter-archipelago scales. Why this should be is unclear, although it may be relevant that many eastern *Samoana* species produce exceptionally sticky mucus [54,57] and this trait could plausibly increase the likelihood of rare inter-island/archipelago phoretic dispersal events, *e.g.*, on avian vectors.

## Conclusions

Isolation is the primary biological characteristic of oceanic islands [9] and our study, placing partulid species diversity into a range-wide phylogenetic context, underlines the critical importance of biotic isolation in the evolution and diversification of this endemic Pacific island tree snail family. Most partulid species diversity – 59 species of *Partula* and 15 species of *Samoana* – is endemic to the Society, Marquesas and Austral archipelagoes. These are the three most isolated archipelagoes in the family’s range, being the most remote from Sahul/Asian continental source populations (Figure 1). Prior to human settlement, they experienced the least impact from continental biotas, *e.g.*, they lacked terrestrial mammals (including bats) and reptiles [71,72]. This is the rarified biotic setting that supported two very distinct generic radiations unsurpassed elsewhere in the range of Partulidae; that of *Partula* being the result of prolonged, uninterrupted cladogenesis within a single archipelago, that of *Samoana* stemming from its ability to disperse and diversify among multiple archipelagoes. Human settlement and modernity/globalization have eroded marine barrier-dependent isolation all across the range of Partulidae, directly exposing these endemic tree snails to an ever growing cast of introduced continental predators. Their continued survival outside of zoos is dependent on our ability to maintain a minimum level of biotic isolation in at least some of the islands of Oceania that could serve as refuges [73] for surviving members of this emblematic Pacific tree snail family.

## Methods

### Sampling

See Table 2 for sampling locations, taxonomic identity and IUCN status of partulid snails genotyped in this study. The vast majority of our samples were obtained from museum collections and/or from captive populations that stemmed from numerous individual collecting events operating over many decades. These specimens were in large part identified by their original collectors, *e.g.*, J.B. Burch identified his extensive 1970 samples of Society Islands partulid samples in the field using Crampton’s taxonomic descriptions [68]. Voucher specimen information is available in the supplemental information together with sampling details for the 662 genotyped partulid snails analyzed in this study (Additional file 3: Table S1).

### Molecular data

Total genomic DNA was isolated using the E.Z.N.A.® Mollusc DNA Kit (Omega Biotech, Norcross, GA) following the manufacturer’s instructions. A 655 nucleotide fragment of the mt cytochrome oxidase I (COI) was amplified with GoTaq DNA Polymerase (Promega, Madison, WI) using the “universal” primer pairs LCO1490/HCO2198 [74]. A 805 (aligned) nucleotide fragment of the large nuclear ribosomal subunit gene (28S) was also amplified from a subset of specimens using D23F/D6R primers [75]. The amplification condition consisted of initial denaturation of 95°C for 2 min, 40 cycles of denaturation (95°C, 30 sec), annealing (45°C for COI and 50°C for 28S, 30 sec), extension (72°C, 1 min), and a final extension at 72°C for 5 min. Amplification products were prepared for cycle sequencing by diluting 1:5 in sterile water. Sequencing was performed in both directions, using the PCR primers, at the University of Michigan DNA Sequencing Core Facility. All DNA sequences obtained have been deposited in GenBank (Additional file 3: Table S1). The resulting chromatograms were edited by comparing both strands using Sequencher 4.8 (Gene Codes Corporation, Ann Arbor, Michigan, USA) and the edited sequences were aligned by eye in Se-Al v2.0a11 [76]. In total, 667 partulids were typed for mt COI and 130 snails for the large nuclear ribosomal subunit gene (28S), yielding 312 and 89 distinct genotypes, respectively.

### Phylogenetic analyses

Maximum parsimony (MP), maximum likelihood (ML) and Bayesian analyses were performed using the partulid genus *Eua* as the outgroup [39]. MP searches were heuristic using PAUP* 4.0b10 [77] with 100 random stepwise additions (MaxTrees set to 10,000) and tree-bisection-reconnection (TBR) branch-swapping. Bootstrap [78] branch support (BS) levels were estimated with 100 replicates (MaxTrees set to 100), 10 random additions each. ML analyses were performed using PAUP* under the best-fit model of nucleotide substitution (COI: K81uf + I + G, 28S: GTR + I + G) selected by the Akaike Information Criterion in 3.7 [79]. The neighbor-joining tree was used as the starting tree with the likelihood parameters found in Modeltest. The branch-swapping algorithm was set to nearest neighbor interchange (NNI) for COI and to TBR for 28S dataset. Bootstrap support values were estimated using a fast-heuristic search with 100 replicates. Bayesian searches were run for 100x10^6^ generations in MrBayes 3.1.2 [80] set for the GTR + Γ + I model. Model parameters were treated as unknown and were estimated for each analysis. Four chains were run simultaneously and trees were sampled every 10,000 cycles. Posterior probability values were estimated by generating a 50% majority rule consensus tree after the burn-in period of 2,500 using PAUP*.

### Time-calibrated BEAST analyses

We chose three time calibration points by identifying within our partulid mt COI Bayesian analysis the most comprehensively sampled, robustly supported clades that were restricted to specific archipelagos or islands of known age. Unsurprisingly, all three concerned Society Islands *Partula* species, by far our best-sampled archipelago and genus. Each calibration was chosen to represent a particular temporal reference point from the geological history of the archipelago. The oldest, Calibration point A (Figure 6), represents the inferred age of the oldest island in the archipelago bearing partulid tree snails: Bora Bora, 4.01-3.21 Mya [81]. The corresponding Eastern *Partula* clade (PPS = 100; Figure 5) includes genotypes from all individuals sampled in the Society Islands and from Rarotonga, which we consider to be a founder population. The next oldest, Calibration point B (Figure 6), represents the inferred age of Raiatea: 2.75-2.44 Mya [82] and the corresponding, well-supported clade (PPS = 97; Figure 5), was exclusively composed of Raiatean taxa. The youngest, Calibration point B (Figure 6), represents the inferred age of Tahiti: 1.12-0.50 Mya [82], the youngest Society Island bearing partulid tree snails. This was applied to the largest Tahiti-exclusive clade (PPS = 99; Figure 5), which probably represents the oldest sampled Tahitian partulid lineage.

We used BEAST 1.7.1 [83] to estimate a time-calibrated partulid phylogeny. This involved employing an uncorrelated lognormal distribution for the molecular clock and the SRD06 Model [84] for codon positions. Normal distributions were used to characterize prior distributions of the clade ages. Two independent MCMC analyses were run for 50 million iterations respectively and sampled every 5000 iterations. Convergence diagnostics were conducted in Tracer v1.5 [85] and reliable ESS values (>200) were ensured. The first 10% trees were discarded as burn-in respectively and the trees were combined in LogCombiner [83]. A maximum credibility tree was generated from the combined trees in TreeAnnotater [83].

### Availability of supporting data

Museum vouchers have been deposited for genotyped snails, DNA sequences have been deposited in GenBank (see Additional file 3: Table S1 for details) and phylogenetic data have been deposited in TreeBase (reference # 16092).

## Additional files

Additional file 1: Figure S1. Bayesian phylogenetic tree showing topological details of the entire Partulidae mt COI dataset. The taxonomic identity of each haplotype is given together with its geographic origin and (for previously published haplotypes) the GenBank reference number. Support levels are shown for each node; from left-to-right: Maximum Parsimony bootstrap support values (>70), Maximum Likelihood bootstrap support values (>70), Bayesian posterior probabilities (>90), respectively. Background details on the genotyped snails are presented in Additional file 3: Table S1. Additional file 2: Figure S2. A time calibrated mt COI BEAST phylogeny of Partulidae showing details of the entire topology. The taxonomic identity of each haplotype is given together with its geographic origin. Date estimates are shown for each node. Within the topology, branches are color-coded for easy reference: green for species of the genus *Eua*, red for species of the genus Samoana, dark blue for Western *Partula* species and light blue for Eastern *Partula* species. Background details on the genotyped snails are presented in Additional file 3: Table S1. Additional file 3: Table S1. Table showing the taxonomic designation, sampling location, shell voucher specimen catalogue number and GenBank Accession number for every partulid genotype employed in this study.
