# Supplementary figures and images for: Evolutionary history of a vanishing radiation: isolation-dependent persistence and diversification in Pacific Island partulid tree snails
Source: BMC Evol Biol. 2014 Sep 24;14:202. doi: 10.1186/s12862-014-0202-3 (PMC4189756; doi:10.1186/s12862-014-0202-3)

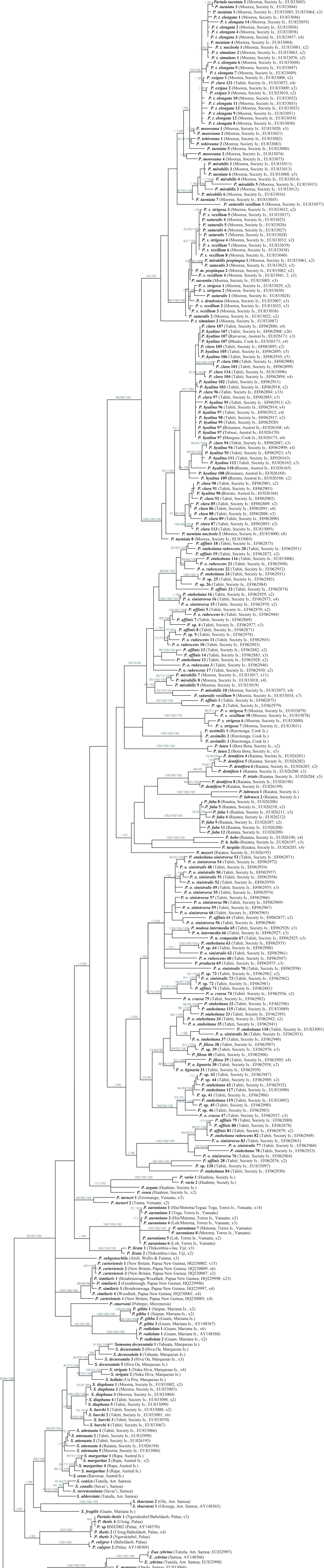

Supplement: Additional file 1: Figure S1. — Bayesian phylogenetic tree showing topological details of the entire Partulidae mt COI dataset. The taxonomic identity of each haplotype is given together with its geographic origin and (for previously published haplotypes) the GenBank reference number. Support levels are shown for each node; from left-to-right: Maximum Parsimony bootstrap support values (>70), Maximum Likelihood bootstrap support values (>70), Bayesian posterior probabilities (>90), respectively. Background details on the genotyped snails are presented in Additional file 3: Table S1. [file 12862_2014_202_MOESM1_ESM.pdf]

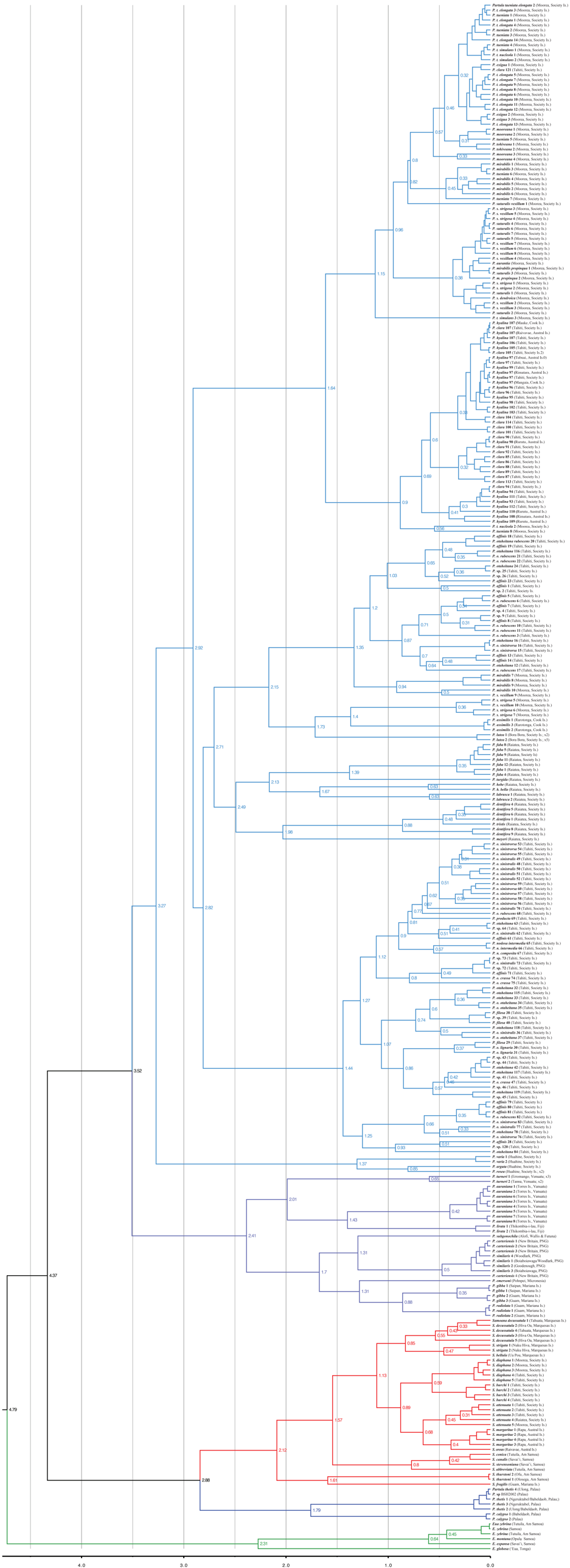

Supplement: Additional file 2: Figure S2. — A time calibrated mt COI BEAST phylogeny of Partulidae showing details of the entire topology. The taxonomic identity of each haplotype is given together with its geographic origin. Date estimates are shown for each node. Within the topology, branches are color-coded for easy reference: green for species of the genus Eua, red for species of the genus Samoana, dark blue for Western Partula species and light blue for Eastern Partula species. Background details on the genotyped snails are presented in Additional file 3: Table S1. [file 12862_2014_202_MOESM2_ESM.pdf]
