# Supplementary material for: Evolutionary history of a vanishing radiation: isolation-dependent persistence and diversification in Pacific Island partulid tree snails
Source: BMC Evol Biol. 2014 Sep 24;14:202. doi: 10.1186/s12862-014-0202-3 (PMC4189756; doi:10.1186/s12862-014-0202-3)
Supplement: Additional file 3: Table S1. — Table showing the taxonomic designation, sampling location, shell voucher specimen catalogue number and GenBank Accession number for every partulid genotype employed in this study. [file 12862_2014_202_MOESM3_ESM.pdf]

Table S1. Taxonomic designation, sampling location, shell voucher specimen catalogue number (UMMZ: University of Michigan Museum of Zoology; MNHN: Muséum National d'Histoire Naturelle, Paris; FMNH: Field Museum of Natural History, Chicago; FLMNH: Florida Museum of Natural History) and GenBank Accession numbers (applied for) for every partulid mt COI haplotype employed in the study and incorporated into the phylogenetic trees shown therein. Museum specimens are indicated in black text, captive zoo specimens are indicated in red text and samples obtained more recently (since 2002) from remnant wild populations are indicated in blue text. Shell voucher numbers were not available for biopsied wild samples, nor for a small number of UMMZ Society Island samples that were damaged during the tissue extraction and lyophilization processes.

Species	Island	Valley Name (if available)	Locality #	Collector, Year	# of snails genotyped for COI	Haplotype #	Voucher number	GenBank Accession #	# of snails genotyped for 28S	GenBank Accession #
Eua globosa Pilsbry & Cooke, 1934	'Eua, Tonga			Burch, 1970	2		UMMZ304357	EU832996[4]	1	KJ424186
E. expansa (Pease, 1872)	Savai'i, Samoa			Price, 1965	1		FMNH152561	EU833069[4]		
E. montana (Cooke & Crampton, 1930)	Upolu, Samoa			Solem & Price, 1965	1		FMNH152750	EU833068[4]		
E. zebrina (Gould, 1847)	Tutuila, Am. Samoa			Burch, 1970	1	2	UMMZ300618	EU832997[4]	1	EU026180[3]
					1	3	UMMZ304358	EU832998[4]		
	Samoa					1		AY148566[6]		
Samoana fragilis (Ferussac, 1821)	Guam, Mariana Is.	Ritidian Pt.		Dybas, 1945	1		FMNH24965	KJ424274	2	KJ424187
S. abbreviata (Mousson, 1869)	Tutuila, Am. Samoa			Burch, 1970	1		UMMZ 304359	KJ424275		
S. conica (Gould, 1847)	Tutuila, Am. Samoa			Burch, 1970	1		UMMZ 304360	KJ424276	1	KJ424188
S. thurstoni (Cooke & Crampton, 1930)	Ofu, Am. Samoa	Mt. Tumu		Price, 1975	1	2	FMNH181109	KJ424277	2	KJ424189
	Olosega, Am. Samoa					1		AY148565[6]		
S. canalis (Mousson, 1865)	Savai'i, Samoa	Asau		Price, 1965	1		FMNH152984	KJ424278		
S. stevensoniana (Pilsbry, 1909)	Savai'i, Samoa	Asau		Price, 1965	1		FMNH152990	KJ424279		
S. margaritae (Crampton & Cooke, 1953)	Rapa, Austral Is.	27.63009°S 144.33535°W (365m)		Fontaine, 2002	1	1	MNHNRp10	KJ424280		
					1	2	MNHNRp10	KJ424281		
		27.62904°S			1	2	MNHNRp9	KJ424281		

		144.33468°W (310m)								
		27.6264°S 144.34257°W (200m)			1	3	MNHNRp4	KJ424282		
		27.61689°S 144.31863°W (200m)			1	4	MNHNRp32	KJ424283		
S. oreas (Crampton & Cooke, 1953)	Raivavae, Austral Is.	23.87589S, 147.69399W (130m)		Fontaine, 2002	1		MNHNRv50	KJ424284		
S. attenuata (Pease, 1864)	Raiatea, Society Is.	Mt. Tefatua (745 m)		Meyer, 2006	1	4	UMMZ300620	EU026194[3]	1	EU026182[3]
	Moorea, Society Is.	Mt. Tohiea Belvedere *Partula* reserve	7	Hickman, 2006	1	5	biopsy	EU833086[4]		
	Tahiti, Society Is.	Tiitauiri R. V.	175	Burch, 1970	1	3	UMMZ300619	EU026193[3]	1	EU026181[3]
		Fareteuira R. V.	198		1	2	UMMZ300427	EU832999[4]		
		Haapupuni V. (115m)		Coote, 2005	1	1	biopsy	EU833066[4]		
S. burchi Kondo, 1973	Tahiti, Society Is.	Taravao Plateau	157	Burch, 1970	2	2	UMMZ300432	EU833001[4]		
			243		2	1	UMMZ300433	EU833000[4]	1	KJ424190
					4	2		EU833001[4]		
		Mt. Atara (1000m)		Coote, 2005	1	3	biopsy	EU833070[4]		
		Mt. Aorai			1	4	biopsy	EU833067[4]	1	KJ424191
S. diaphana (Crampton & Cooke, 1953)	Moorea, Society Is.	Hotutea V.	279	Burch, 1970	2	1	UMMZ300548	EU833002[4]		
					1	2		EU833003[4]		
					1	3		EU833004[4]		
	Tahiti, Society Is.	Mt. Aorai, (1300m)		Coote, 2006	1	4	biopsy	EU833098[4]		
		Mt. Aorai (1157m)		Coote, 2007	1		biopsy			
		Mt. Pihaaiateta		Holland, 2004	1	5	biopsy	EU833099[4]		
S. bellula (Hartman, 1885)	Ua Pou, Marquesas Is.	Pua Maka (~500m)		Holland, 2005	1		UMMZ304361	KJ424285		
S. decussatula (Pfeiffer, 1850)	Hiva Oa, Marquesas Is.	Mt. Temetiu (900m)		Holland, 2004	1	3	biopsy	KJ424288		
		Mt. Temetiu (938m)			1	3	biopsy			
		Mt. Temetiu (950m)			1	3	biopsy			
		Mt. Temetiu (1100m)			1	5	biopsy	KJ424290		

		Mt. Temetiu (900m)		Coote, 2005	1	2	UMMZ304362	KJ424287	1	KJ424192
	Tahuata, Marquesas Is.	Vaitahu		1995	1	1	UMMZ304363	KJ424286	1	KJ424193
		Uuoa			1	4	UMMZ304364	KJ424289	1	KJ424194
S. strigata (Pease, 1868)	Nuku Hiva, Marquesas Is.	Mt. Tekoa, 8°52.246S, 140°10.378W		Holland, 2004	4	1	biopsy	KJ424291		
					1	2		KJ424292		
Partula calypso Semper, 1865	Babeldaob, Palau			Rundell, 2005	1	1	FMNH310532	KJ424293	1	KJ424195
	Palau					2		AY148569[6]		
P. thetis Semper, 1865	Ulong, Palau			Crombie, 1995	2	2	FLMNH252892	KJ424296	3	KJ424197
					1	4		KJ424299		
	Ngeruktabel, Palau			Kraus, 1998	2	1	FLMNH271887	KJ424294	3	KJ424196
					1	3		KJ424298		
	Babeldaob, Palau			Rundell, 2006	1	1	FMNH308162	KJ424295	1	KJ424198
					2	2	FMNH308163	KJ424297		
	Palau					1		AY148570[6]		
P. gibba Ferussac, 1821	Saipan, Mariana Is.			Burch, 1970	2	1	UMMZ304365	KJ424300	1	KJ424199
				Burch, 1970	2	1	UMMZ304366	KJ424301		
	Guam, Mariana Is.	Mt. Lamlam		Krauss, 1958	1	2	FMNH77825	KJ424302	1	KJ424200
						3		AY148567[6]		
P. radiolata Pfeiffer, 1846	Guam, Mariana Is.			Kraus, 1995	2	1	FLMNH281059	KJ424303	2	KJ424201
P. radiolata Pfeiffer, 1846	Guam, Mariana Is.			Kraus, 1995	2	1	FLMNH280973	KJ424303	2	KJ424201
					2	1	FLMNH280968			
P. emersoni Pilsbry, 1913	Pohnpei, Micronesia	Navy Hill		1993	2	2	UMMZ304367	KJ424304	1	KJ424202
						1		AY148568[6]		
		Mt. Kupwuriso		Holland, 2011	1		Biopsy	KJ424185		
P. carteriensis (Quoy & Gaimard, 1832)	New Britain, Papua New Guinea	Pomio, 5.5183°S 151.5067°E		Slapcinsky, 2005	7	1	FLMNH366477	HQ230002[5]	1	KJ424203
P. carteriensis (Quoy & Gaimard, 1832)	New Britain, Papua New Guinea	Pomio, 5.5183°S 151.5067°E		Slapcinsky, 2005	3	2		HQ230009[5]		KJ424203
					1	3	FLMNH366477	HQ230007[5]	1	
P. similaris Hartman, 1886	Goodenough, Papua New Guinea	Pomio, 5.5153°S 151.5111°E		Burch, 1966	1	4	FLMNH366510	HQ230005[5]	1	KJ424204
					7	1		HQ230004[5]	2	KJ424205
		Marmar, 5.5173°S 151.5007°E			1	1	FLMNH366509	HQ230003[5]	1	KJ424206

		Marmar, 5.5173°S 151.5007°E			3	2		HQ230010[5]	1	KJ424206
					1	3	FLMNH366509	HQ230008[5]		
					3	4	UMMZ302228	HQ230006[5]		
					1	2		HQ229996[5]		
P. similaris Hartman, 1886 *P. auraniana* Hartman, 1888	Boiaboiawaga, Papua New Guinea	10°12.7'S, 150°54.2'E		Kraus, 2002	9	1	FLMNH303716	HQ229998[5]		
	Boiaboiawaga,				4	3		HQ229997[5]	2	KJ424207
	Papua New Guinea Woodlark, Papua New Guinea	10°12.7'S, 150°54.2'E Guasapa, 9.13444°S 152.56637°E		Kraus, 2002 Slapcinsky, 2003	8	1	FLMNH303716 FLMNH303731	HQ229999[5]	3	KJ424208
	Woodlark,	Guasapa, 9.13444°S		Slapcinsky,	4	4		HQ230001[5]		
	Papua New Guinea Hiu, Vanuatu	152.56637°E Guasapa, 9.2241°S, 152.944°E		2003 Fontaine, 2009	6	1	FLMNH303731 FLMNH333131	HQ230000[5]	3 2	KJ424208 KJ424209
					4	1	Biopsy	KJ424305	1	KJ424212
P. auraniana Hartman, 1888	Hiu, Vanuatu				1	3	Biopsy	KJ424310		
	Loh, Vanuatu				2	4	Biopsy	KJ424312	1	KJ424213
P. turneri Pfeiffer, 1860	Loh, Vanuatu				2	5		KJ424314		
	Metoma,				1	6	Biopsy	KJ424315	1	KJ424213
	Vanuatu				1	1	Biopsy	KJ424306	1	KJ424214
	Metoma,				1	3		KJ424311		
	Vanuatu			Fontaine,	1	4		KJ424313		
	Tegua, Vanuatu			2009	1	7	Biopsy	KJ424316	1	KJ424214
				Price, 1972	1	8	Biopsy	KJ424317	1	KJ424210
					5	1		KJ424307		
	Toga, Vanuatu				4	1	Biopsy	KJ424308	1	KJ424211
	Toga, Vanuatu				1	2		KJ424309		
	Erromango, Vanuatu	Ipota			3	1	Biopsy FMNH175385	KJ424318	1	KJ424215
P. turneri Pfeiffer, 1860	Tanna, Vanuatu	Yeruareng		Huck, 1984	2	2	FLMNH58233	KJ424319		
P. lirata Mousson, 1865	Thikombia-i-lau, Fiji			1999	3	1	UMMZ304368	KJ424320	4	KJ424216
P. lirata Mousson, 1865	Thikombia-i-lau, Fiji			1999	2	2	UMMZ304368	KJ424321	4	KJ424216
P. subgonochila Mousson, 1871	Alofi, Wallis & Futuna			2007	3		UMMZ304369	KJ424322	3	KJ424217

P. assimilis Pease, 1868	Ratotonga,<br>Cook Is.	Takuraine		Coote, 2004	1	1	UMMZ304370	KJ424323	1	KJ424218
P. assimilis Pease, 1868	Ratotonga,				1	2		KJ424324		
P. lutea Lesson, 1831	Cook Is.	Takuraine		Coote, 2004	1	3	UMMZ304370	KJ424325	1	KJ424218
	Bora Bora,<br>Society Is.			Burch, 1970	2	1	UMMZ304371	KJ424326	1	KJ424219
P. lutea Lesson, 1831	Bora Bora,				5	2		KJ424327		
P. dentifera Pfeiffer,<br>1854	Society Is.<br>Raiatea, Society<br>Is.	Haava V.		Burch, 1970<br>Burch, 1970	3	1	UMMZ304371<br>UMMZ300625	EU026200[3]	1<br>1	KJ424219<br>EU026187[3]
P. dentifera Pfeiffer,<br>1854	Raiatea, Society<br>Is.	Vaiaau V.			1	4	UMMZ300626	EU026201[3]	2	EU026188[3]
P. faba (Gmelin, 1791)	Raiatea, Society<br>Is.			Burch, 1970	1	5	UMMZ300627	EU026202[3]		
		Vaiaau V.		1991	2	6	UMMZ300628	EU026203[3]	2	EU026188[3]
		Hamoa			1	8	UMMZ300624	EU026198[3]	4	EU026186[3]
		Hamoa			1	9		EU026199[3]		
		Valley in Tupua<br>Bay		1991<br>Burch, 1970	3	5	UMMZ300624<br>UMMZ300632	EU026210[3]	4	EU026186[3]
P. faba (Gmelin, 1791)	Raiatea, Society	Vaiaau V.			3	1	UMMZ300633	EU026211[3]	1	EU026192[3]
P. hebe (Pfeiffer, 1846)	Is.	Vaiaau V.		Burch, 1970	1	4	UMMZ300633	EU026212[3]	1	EU026192[3]
	Raiatea, Society<br>Is.	Hamoa		1991-2	1	8	UMMZ300631	EU026206[3]	1	EU026191[3]
					2	9		EU026207[3]		
		Hamoa		1991-2	1	11	UMMZ300631	EU026208[3]		
		Vaiaau V.		Burch, 1970	1	12	UMMZ300622	EU026209[3]		
					4			EU026196[3]	1	EU026184[3]
P. hebe bella Pease,<br>1881	Raiatea, Society<br>Is.	Hotopuu		1991	3		UMMZ300623	EU026197[3]	3	EU026185[3]
P. labrusca Crampton &<br>Cooke, 1953	Raiatea, Society<br>Is.			1992	1	1	UMMZ304372	KJ424328		
P. labrusca Crampton &<br>Cooke, 1953	Raiatea, Society<br>Is.				1	2		KJ424329		
P. meyeri Burch, 2007	Raiatea, Society<br>Is.	Mt. Tefatua (950 m)		1992<br>Meyer, 2006	1		UMMZ304372<br>UMMZ300621	EU026195[3]	1	EU026183[3]
P. tristis Crampton &<br>Cooke, 1953	Raiatea, Society<br>Is.	Tevaitoa		1991	5		UMMZ300629	EU026204[3]	2	EU026189[3]
P. turgida (Pease, 1865)	Raiatea, Society<br>Is.			1991	4		UMMZ300630	EU026205[3]	2	EU026190[3]
P. arguta (Pease, 1865)	Huahine,<br>Society Is.			1991	1		UMMZ304373	KJ424330		
P. rosea Broderip, 1832	Huahine,			1987	2		UMMZ304374	KJ424331	1	KJ424220

	Society Is.									
P. varia Broderip, 1832	Huahine, Society Is.			1991, 1994	1	1	UMMZ304375	KJ424332	1	KJ424221
P. varia Broderip, 1832	Huahine, Society Is.			1991, 1994	1	2	UMMZ304375	KJ424333		
P. aurantia Crampton, 1932	Moorea, Society Is.	Faamaariri V. (300m)	270	Burch, 1970	3		UMMZ300516, 300517	EU833005[4]	1	KJ424222
P. exigua Crampton, 1917	Moorea, Society Is.	Faamaariri V. (150m)	269	Burch, 1970	2	2	UMMZ300512	EU833009[4]		
P. exigua Crampton, 1917	Moorea, Society Is.	Faamaariri V. (300m)	270		2	3	UMMZ300518	EU833010[4]	1	KJ424223
P. mirabilis Crampton, 1924	Moorea, Society Is.	Puutu V. (400m)	272	Burch, 1970	2	1	UMMZ300525	EU833008[4]		
		Southern slope of Mt. Rotui, Matapoopoo V. (150-200m)	273	Burch, 1970	1	1	UMMZ300534	EU833011[4]		
P. mirabilis Crampton, 1924	Moorea, Society Is.				1	2	UMMZ300531	EU833012[4]		
					1	3	UMMZ300535	EU833013[4]		
P. mirabilis propinqua Crampton, 1932	Moorea, Society Is.	Southern slope of Mt. Rotui, Matapoopoo V. (150-200m)	273		1	4	UMMZ300531	EU833014[4]		
					1	5	UMMZ300528	EU833015[4]		
					1	6	UMMZ300533	EU833016[4]		
		Mouaroa V. (200-300m)	275	Burch, 1970 1984-5	11	7	UMMZ300490, 300491, 300526, 300529	EU833017[4]		
					4	8	UMMZ300492, 300555	EU833018[4]	1	KJ424224
		Mouaroa V. (200-300m)	275		1	9	UMMZ300552	EU833019[4]	1	KJ424224
		Fareaito V. ?	11 ?		4	10	UMMZ301016	EU833075[4]		
		Matapoopoo V. (150-250m)	273	Burch, 1970	2	1	Shell voucher not available	EU833061[4]	1	KJ424225
P. mirabilis propinqua Crampton, 1932	Moorea, Society Is.	Matapoopoo V. (150-250m)	273	Burch, 1970	2	2	Shell voucher not available	EU833062[4]	1	KJ424225
P. mooreana Hartman, 1880	Moorea, Society Is.	Hotutea V. northeastern ridge (480m)	278	Burch, 1970	1	1	UMMZ300547	EU833020[4]		
P. mooreana Hartman, 1880	Moorea, Society Is.	Hotutea V. northeastern ridge	278	Burch, 1970	1	2	UMMZ300546	EU833021[4]		
P. suturalis Pfeiffer,	Moorea, Society	(480m)	279	1985	4	1	Shell voucher not available	EU833020[4]	1	KJ424226

1855	Is.	Hotutea V. northeastern ridge (620m)								
		Maatea V.	12		1	4	UMMZ301017	EU833073[4]	1	KJ424227
		Atimaha Ridge	13	1982	1	3	UMMZ301018	EU833074[4]	1	KJ424228
		Faamaariri V. (300m)	270	Burch, 1970	2	2	UMMZ300519	EU833022[4]		
P. suturalis Pfeiffer, 1855	Moorea, Society Is.	Puutu V. (250m)	271		3	3	UMMZ300521-300523	EU833023[4]		
P. s. dendroica Crampton, 1924	Moorea, Society Is.	Hotutea V. (620-700m)	279		1	1	UMMZ300557	EU833024[4]		
		Hotutea V. (620-700m)<br>Matapoopoo V. (400m)	279<br>274	Burch, 1970<br>Burch, 1970	1	4	UMMZ300557<br>UMMZ300537	EU833025[4]		
					1	5		EU833026[4]		
					1	6		EU833027[4]		
					1	7		EU833028[4]		
					3			EU833007[4]	1	KJ424229
P. s. strigosa Pfeiffer, 1856	Moorea, Society Is.	Hotutea V. (400-480m)	276	Burch, 1970	2	1	UMMZ300556	EU833029[4]		
P. s. strigosa Pfeiffer, 1856	Moorea, Society Is.	Hotutea V. (400-480m)	276		1	2	UMMZ300556<br>UMMZ300543	EU833030[4]		
					1	7		EU833031[4]		
P. s. vexillum Pease, 1866	Moorea, Society Is.	Hotutea V. (480-620m)	278	Burch, 1970<br>1980	1	3		EU833032[4]		
		Hotutea V. (620m)	279		1	3	UMMZ300558	EU833032[4]	1	KJ424230
		Hotutea V. (620m)	279		2	4	UMMZ300558	EU833033[4]	1	KJ424230
		Maatea V.	12		1	5	UMMZ301020	EU833079[4]	1	KJ424231
		Maatea V.		1985	1	6	UMMZ301019	EU833080[4]	1	KJ424232
		Faatoai V. northern slope of Mt. Tautuapae (350m)	12<br>261	Burch, 1970	7	9	UMMZ300487	EU833034[4]	1	KJ424233
P. s. vexillum Pease, 1866	Moorea, Society Is.	Paparoa V. (100m)	267		2	2	UMMZ300494	EU833035[4]	1	KJ424234
					1	3	UMMZ300501	EU833036[4]		
P. taeniata (Mörch, 1850)	Moorea, Society Is.	Paparoa V. (100m)<br>Paparoa V. (350m)	267<br>268		1	2	UMMZ300504, 300505, 300507, 300509	EU833035[4]		
				Burch, 1970<br>1982	1	5	UMMZ300504, 300505, 300507, 300509	EU833037[4]		
		Paparoa V. (350m)	268		1	6		EU833038[4]		
		Mouaroa V. (200-300m)	275		1	7		EU833039[4]		
					1	8	UMMZ300538, 300539, 300554	EU833040[4]		
					4	4		EU833041[4]		

		Hotutea V. (400-480m)	276		1	4	UMMZ300559	EU833042[4]		
		Fareaito V.	11		1	1	UMMZ301021	EU833077[4]	1	KJ424235
		Vaianai V.	14	1985	1	10	UMMZ301022	EU833078[4]	1	KJ424236
		Southern slope of Mt. Rotui, Matapoopoo V. (150-250m)	273	Burch, 1970	1	2	UMMZ300527, 300532	EU833043[4]		
P. taeniata (Mörch, 1850)<br>*P. t. elongata* Pease, 1866	Moorea, Society Is.<br>Moorea Society Is.	Southern slope of Mt. Rotui, Matapoopoo V. (150-250m)<br>Maatea V. (180m)	273<br>1	Burch, 1970<br>Coote, 2005, 2006	1	3	UMMZ300527, 300532<br>biopsy	EU833044[4]		
					1	7		EU833045[4]		
					2	1		EU833085[4]		
		Haumi V.	2	Coote, 2005	1	1	biopsy	EU833064[4]		
		Moruu V.	3	Coote, 2006	1	8	biopsy	EU833065[4]	1	KJ424237
		Morioahu V. (243m)	4	Coote, 2007	1	5	biopsy	EU833088[4]		
		Opunohu Bay	5	Hickman, 2006	5	6	biopsy	EU833071[4]	1	KJ424238
		Mt. Tohiea (1150m) 17°33'04S 149°49'18W	6	Meyer, 2002	1	4	biopsy	EU833084[4]		
		Paparoa V. (100m)	267	Burch, 1970	1	1	UMMZ300496, 300498, 300499, 300500, 300502, 300503	EU833046[4]		
P. t. elongata Pease, 1866<br>*P. t. nucleola* "Pease" Schmeltz, 1874	Moorea Society Is.<br>Moorea, Society Is.	Paparoa V. (100m)<br>Paparoa V. (300m)	267<br>268	Burch, 1970<br>Burch, 1970	1	5	UMMZ300496, 300498, 300499, 300500, 300502, 300503<br>UMMZ300506, 300508	EU833047[4]		
					1	6		EU833048[4]		
					1	7		EU833049[4]		
					1	8		EU833050[4]		
					1	9		EU833051[4]		
					1	10		EU833052[4]		
					1	11		EU833053[4]		
					1	12	UMMZ300506, 300508	EU833054[4]		
		Paparoa V. (300m)<br>Mouaroa V. (200-300m)	268<br>275		1	13		EU833055[4]		
					1	2	UMMZ300488, 300540, 300542, 300551, 300553	EU833056[4]		
		Mouaroa V. (200-	275		4	3	UMMZ300488,	EU833057[4]	1	KJ424239

		300m) Faatoai V.	261		1	4	300540, 300542,	EU833058[4]		
					1	14	300551, 300553	EU833059[4]		
					8	2	UMMZ300489	EU833060[4]	1	KJ424240
P. t. nucleola "Pease" Schmeltz, 1874	Moorea, Society Is.	Faatoai V.	15	1981	2	1	UMMZ301023	EU833081[4]	1	KJ424241
P. t. simulans Pease, 1866	Moorea, Society Is.	Hotutea V. (400-480m)	276	Burch, 1970	2	2	UMMZ300544	EU833063[4]	1	KJ424242
P. t. simulans Pease, 1866	Moorea, Society Is.	Haapiti V.	16	1986	2	1	UMMZ301024, 301025	EU833076[4]	1	KJ424243
P. tohiveana Crampton, 1924	Moorea, Society Is.	Mt. Tohiea (1150m) 17°33'04S 149°49'18W	6	Meyer, 2006	1	3	biopsy	EU833087[4]		
		Fareaito V.	11	1982	1	1	UMMZ301026	EU833082[4]	1	KJ424244
P. tohiveana Crampton, 1924	Moorea, Society Is.				1	2	UMMZ301027	EU833083[4]	1	KJ424245
		Fareaito V.	11	1982						
P. affinis Pease, 1867	Tahiti, Society Is.	Afeu R. V.	107	Burch, 1970	2	61	UMMZ300363	EF062877[1]		
P. affinis Pease, 1867	Tahiti, Society Is.	Tirahi R. V.	139		2	28	UMMZ300409	EF062876[1]	1	KJ424246
P. clara Pease, 1864	Tahiti, Society Is.	Vaitepiha V.	142		1	1	UMMZ300411	EF062875[1]		
		Vaitehoro R. V	152		1	18	UMMZ300417	EF062873[1]		
		Vaitehoro R. V	152		2	19	UMMZ300417	EF062872[1]	1	KJ424247
		Ahaavini V.	161		1	23	UMMZ300441, 300442	EF062874[1]		
		Tiitauiri R. V	174		1	80	UMMZ300452	EF062878[1]		
		Tiitauiri R. V	176	Burch, 1970	1	79	UMMZ300460	EF062880[1]		
			176	1995	2	81	UMMZ300457	EF062879[1]	1	KJ424248
		Tereia R. V.	178		1	71	UMMZ300464	EF062881[1]		
		Tetiairiroa V	192		2	5	UMMZ 300467, 300470	EF062870[1]		
		Fareteuira R. V.	198		1	8	UMMZ300476	EF062871[1]		
		Tuauru R. V.	211		1	7	UMMZ300482	EF062869[1]		
		Te Pari District	3		2	13	UMMZ300560, 300561	EF062882[1]		
		Te Pari District	3	1995	3	14	UMMZ300561	EF062883[1]		
		Moaroa R. V.	120	Burch, 1970	26	96	UMMZ300382, 300383, 300386, 300388	EF062892[1]		
P. clara Pease, 1864	Tahiti, Society Is.	Taharuu R. V.	125	Burch, 1970	2	87	UMMZ300393	EF062893[1]		
P. filosa Pfeiffer, 1853		Toheimahu R. V	135	1995	1	96	UMMZ300396,	EF062884[1]		

	Tahiti, Society Is.						300398			
		Tirahi R. V.	136		3	94	UMMZ300401	EF062887[1]		
		Vaitepiha V.	142		4	107	UMMZ300412	EF062886[1]		
		Vaitehoro R. V.	152		2	97	UMMZ300435	EF062885[1]		
		Vairaharaha R. V.	165		6	86	UMMZ300426	EF062891[1]	1	KJ424249
		Tahiria R. V.	169		1	85	UMMZ300454	EF062889[1]		
			169		2	88	UMMZ300453	EF062888[1]		
		Tahiria R. V.	170		1	85	UMMZ300456	EF062889[1]		
					1	89	UMMZ300456	EF062890[1]		
		Paihau R. V.	170<br>224		4	104	shell voucher unavailable	EF062894[1]	1	KJ424250
		Te Pari District	3		4	96	UMMZ300562	EF062904[1]		
		Papenoo-Maroto V.	1	1997	2	90	UMMZ300563	EF062901[1]		
					1	91		EF062903[1]		
		Papenoo-Maroto V.	1	1997	1	92	UMMZ300563	EF062902[1]		
		Onoheha-Tefaaiti V.	5	Coote, 2004	1	105	biopsy	EF062896[1]		
		Vaipahi R. V.	11	Coote, 2004	1	96	biopsy	EF062897[1]		
		Faarapa V.	3	Coote, 2005	1	105	biopsy	EF062895[1]		
		Taapua V.	12		1	97	biopsy	EF062898[1]		
		Maruapo V. (120m)	14	Coote, 2005	1	100	biopsy	EF062900[1]		
		Papehue R. V. (175m)	13	Coote, 2006	1	101	Biopsy	EF062899		
		Tiapa V. (140m)		Coote, 2006	4	121	biopsy	EU833072[4]	1	KJ424251
		Taapeha V. (<30)		Coote, 2007	1	96	biopsy	EU833094		
		Fautaua V. (490m)			1	113	biopsy	EU833095[4]		
		Vaipoe V. (272m)		Coote, 2007	1	114	biopsy	EU833096[4]		
		Pirae V.	200	Burch, 1970	4	29	UMMZ300428, 300429	EF062905[1]		
P. filosa Pfeiffer, 1853	Tahiti, Society Is.				1	38	UMMZ300479	EF062907[1]		
P. hyalina Broderip, 1832	Tahiti, Society Is.	Pirae V.	200	Burch, 1970	1	40	UMMZ300479	EF062906[1]		
		Orofero V.	99	Burch, 1970	3	112	UMMZ300360	EU026162[2]		
P. hyalina Broderip, 1832	Tahiti, Society Is.	Afeu R. V.	107		4	96	UMMZ300367	EF062914[1]		
		Mapuaura R. V.	131		3	97	UMMZ300395	EF062912[1]		
P. nodosa composite Crampton, 1917	Raivavae, Ausral Is.	Tirahi R. V.	138		2	95	UMMZ300407	EF062913[1]		
		Fautaua R. V.	144	Burch, 1970<br>1996	11	107	UMMZ300413	EF062908[1]		
		Fautaua R. V.	220		8	107	shell voucher unavailable	EF062908[1]		
		Tetiairiroa V.	222		2	94	shell voucher	EF062909[1]	1	KJ424252

							unavailable			
			222<br>192		7	107	shell voucher unavailable	EF062908[1]		
					1	102	UMMZ300471	EF062911[1]		
		Tuauru R. V	211		4	106	UMMZ300483	EF062910[1]	1	KJ424253
		Tipaerui V.	217		5	105	shell voucher unavailable	EF062915[1]		
		Tahaute V.	2		4	93	UMMZ300564	EF062924[1]		
		Onoheha-Tefaaiti V.	6	Coote, 2004	1	103	biopsy	EF062918[1]		
		Haapupuni V.	4	Coote, 2005	2	98	biopsy	EF062917[1]		
		Tahaute V.	7		1	103	biopsy	EF062919[1]		
		Vaitoare R. V.	8		1	99	biopsy	EF062920[1]		
		Raroui R. V.	9	Coote, 2005	1	94	biopsy	EF062921[1]		
		Raroui R. V.	9	Coote, 2006	1	97	biopsy	EF062922[1]		
		Matatia V. (120m)	15		1	93	biopsy	EF062923[1]		
		Ahonu R. V.	2		1	106	biopsy	EF062916[1]		
		Tihiute R. V. (120m)	18	Coote, 2006	1	111	biopsy	EU026163[2]		
		Ahaavini V. (90m)		Coote, 2007	1	94	biopsy	EU833093[4]		
		23°85709S, 147°61591W (20m)		Fontaine & Gargominy, 2002	2	107	MNHNRv39	EU026171[2]	1	KJ424254
	Raivavae, Ausral Is.	23°87467S, 147°68989W (90m)		Fontaine & Gargominy, 2002	1	107	MNHNRv71	EU026172[2]		
	Rimatara, Austral Is.	22°63868S, 152°80645W (10m)		Fontaine & Gargominy, 2004	4	97	MNHNRm31	EU026168[2]	1	KJ424255
					1	108		EU026169[2]		
	Rimatara, Austral Is.<br>Rurutu, Austral Is.	22°63868S, 152°80645W (10m)<br>Mato Arei, 22°45687S, 151°32423W (10m)		Fontaine & Gargominy, 2004<br>Fontaine & Gargominy, 2003	1	109	MNHNRm31<br>MNHNRr06	EU026166[2]		
	Rurutu, Austral Is.	S. Paparai, 22°50772S, 151°33412W (60m)		Fontaine & Gargominy, 2003	1	109	MNHNRr36	EU026167[2]		
	Tubuai, Austral Is.	Falises de Matonaa (<10m)		Coote, 2005	1	90	UMMZ300610	EU026164[2]	1	KJ424256

		Pte Arei, Peva (<20m)		Coote, 2005 Fontaine & Gargominy, 2003	1	110	UMMZ300611	EU026165[2]	1	KJ424257
		23°38198S, 149°52343W (2m)			1	97	MNHNTb11	EU026170[2]		
	Mangaia, Cook Is.			McCormack, 2006	6	97	UMMZ300613-300617	EU026175-026179[2]		
	Mauke, Cook Is			Coote, 2007	4	107	UMMZ300612	EU026173, 026174[2]		
	Tahiti, Society Is.	Punaruu R. V.	91	Burch, 1970	3	67	shell voucher unavailable	EF062925[1]	1	KJ424258
P. n. intermedia Crampton, 1917	Tahiti, Society Is.	Papehue R. V.	44	1984	3	65	UMMZ300572	EF062926[1]	1	KJ424259
P. n. intermedia Crampton, 1917	Tahiti, Society Is.	Papehue R. V.	44	1984	3	66	UMMZ300565,300566	EF062927[1]		
P. otaheitana (Brugière, 1789)	Tahiti, Society Is	Punaruu R. V.	94	Burch, 1970	2	16	UMMZ300359	EF062929[1]		
P. otaheitana (Brugière, 1789)	Tahiti, Society Is	Moaroa R. V.	120		1	116	UMMZ300386	EU833006[4]		
P. o. crassa Pease, 1884	Tahiti, Society Is.	Tirahi R. V.	138	Burch, 1970	2	12	UMMZ300406, 300408	EF062928[1]		
		Mt. Marau, Belevedere	5	1995	1	42	UMMZ300567	EF062932[1]		
		Mt. Marau, Belevedere	5	1995	1	78	UMMZ300567	EF062933[1]	1	KJ424260
					1	63	UMMZ300570	EF062935[1]		
		Mt. Aorai, Fare Mato	1	Coote, 2005	1	84	biopsy	EF062930[1]	1	KJ424261
		Mt. Atara, Taravao Plateau (1050m)	10	Coote, 2005	1	24	biopsy	EF062931[1]	1	KJ424262
		Mt. Aorai, Fare Mato (1300m)	1	Coote, 2006	1	32	biopsy	EF462396[1]		
		Mt. Aorai, Fare Mato (1400m)	1	Coote, 2006	1	33	biopsy	EF462395[1]		
		Mt. Aorai, Fare Mato (1402m)	1	Coote, 2007	1	115	biopsy	EU833089[4]		
		Mt. Marau (1246m)			1	117	biopsy	EU833090[4]		
		Mt. Marau (1246m)		Coote, 2007	1	118	biopsy	EU833091[4]		
		Mt. Marau (1391m)		Burch, 1970	1	119	biopsy	EU833092[4]		
		Punaruu R. V	90		3	47	UMMZ300357	EF062937[1]	1	KJ424263
P. o. crassa Pease, 1884	Tahiti, Society Is.	Orofero V.	99	Burch, 1970	1	75	UMMZ300361	EF062982[1]	1	KJ424264
		Paihau R. V.	224	Burch, 1970	2	74	shell voucher	EF062936[1]	1	KJ424265

P. o. lignaria Pease, 1865	Tahiti, Society Is.						unavailable			
		Tipaerui V.	217		2	30	UMMZ300430	EF062938[1]		
P. o. lignaria Pease, 1865	Tahiti, Society Is.	Tipaerui V.	217	Burch, 1970	1	31	UMMZ300431	EF062939[1]	1	KJ424266
P. o. otaheitana (Brugière, 1789)	Tahiti, Society Is	Fautaua R. V.	144	Burch, 1970	1	35	UMMZ300414	EF062941[1]	1	KJ424267
P. o. otaheitana (Brugière, 1789)	Tahiti, Society Is		144		2	37	UMMZ300414	EF062940[1]		
		Fautaua R. V.	221	Burch, 1970	2	34	UMMZ300484	EF062942[1]		
P. o. rubescens Reeve, 1850	Tahiti, Society Is	Toheimahu R. V.	135	Burch, 1970	1	68	UMMZ300397	EF062947[1]		
P. o. rubescens Reeve, 1850	Tahiti, Society Is	Taravao Plateau	157		2	17	UMMZ300440	EF062950[1]		
		Taravao Plateau	243		1	20	UMMZ300434	EF062951[1]		
P. o. sinistralis Pease, 1884	Tahiti, Society Is	Ahaavini V.	159		1	21	UMMZ300419, 300421-300424	EF062948[1]		
		Ahaavini V.	159	Burch, 1970	1	82	UMMZ300419, 300421-300424	EF062949[1]		
		Onoheha V.	186	Coote, 2005	1	3	UMMZ300425	EF062946[1]		
		Tetiairiroa V	192		1	6	UMMZ300468	EF062944[1]		
		Tetiairiroa V	192		1	10	UMMZ300469	EF062943[1]	1	KJ424268
					1	11	UMMZ300468	EF062945[1]		
		Mt. Atara, Taravao Plateau (1050m)	10		1	22	biopsy	EF062952[1]		
		Temarua V.	108	Burch, 1970	3	49	UMMZ300368, 300369	EF062955[1]		
P. o. sinistralis Pease, 1884	Tahiti, Society Is	Temarua V.	110	Burch, 1970	1	50	UMMZ300372	EF062957[1]		
			110		1	51	UMMZ300372	EF062956[1]		
P. o. sinistrorsa Pease, 1884	Tahiti, Society Is.		113		1	70	UMMZ300376	EF062958[1]		
					1	52		EF062959[1]		
		Papeiti R.	113	Burch, 1970	1	77	UMMZ300376	EF062960[1]		
			123		1	62	UMMZ300377	EF062961[1]		
					1	48	UMMZ300389, 300390	EF062954[1]	1	KJ424269
		Tereia R. V.	180		1	73	UMMZ300465	EF062962[1]		
		Fautaua R. V.	221		1	36	UMMZ300485	EF062953[1]		
		Afeu R. V.	106		1	83	UMMZ300362	EF062963[1]		
P. o. sinistrorsa Pease, 1884	Tahiti, Society Is.	Afeu R. V.	107	Burch, 1970	1	76	UMMZ300365, 300366	EF062964[1]		
P. producta Pease, 1865	Tahiti, Society Is.	Faurahi R. V.	117	Burch, 1970	2	15	UMMZ300378	EF062970[1]		
		Faurahi R. V.	117		1	58	UMMZ300378	EF062969[1]	1	KJ424270
		Moaroa R. V.	120		1	53	UMMZ300385,	EF062971[1]		

							300387			
		Moaroa R. V.<br>Taharuu R. V.	120<br>125		1	54	UMMZ300385, 300387	EF062972[1]		
					4	16	UMMZ300391, 300393	EF062973[1]		
		Taharuu R. V.<br>Tahiria R. V.	125<br>169		1	55	UMMZ300391, 300393	EF062974[1]		
					1	57	UMMZ300451	EF062966[1]		
		Tahiria R. V.<br>Faurahi R. V.	169		1	60	UMMZ300450	EF062965[1]		
			170		1	56	UMMZ300455	EF062968[1]		
			170		1	59	UMMZ300455	EF062967[1]		
			117		3	69	UMMZ300379	EF062975[1]	1	KJ424271
P. sp.	Tahiti, Society Is.	Punaruu R. V	94	Burch, 1970	1	46	UMMZ300358	EF062983[1]		
P. sp.	Tahiti, Society Is.	Vaiarava R. V.	162	Burch, 1970<br>Coote, 2005	1	2	UMMZ300443	EF062979[1]		
		Tereia R. V.	178		1	72	UMMZ300461	EF062981[1]		
		Tereia R. V.<br>Fareteuira R. V.	178<br>198		2	73	UMMZ300462, 300463	EF062980[1]		
					3	4	UMMZ300474, 300475	EF062977[1]		
		Fareteuira R. V.<br>Pirae V.	198<br>200		1	9	UMMZ300474	EF062978[1]		
					3	39	UMMZ300477	EF062976[1]	1	KJ424272
		Mt. Atara, Taravao Plateau (1050m)	10		1	25	biopsy	EF062985[1]		
		Mt. Atara, Taravao Plateau (1050m)<br>Mt. Marau, Site 1	10<br>16	Coote, 2005<br>Coote, 2007	1	26	biopsy	EF062984[1]		
					2	44	biopsy	EF062989[1]		
		Mt. Marau, Site 1<br>Mt. Marau, Belvedere	16<br>17		1	45	biopsy	EF062990[1]		
					1	64	biopsy	EF062988[1]		
					1	41	biopsy	EF062986[1]		
		Mt. Marau, Belvedere<br>Pointe Terurua	17		1	43	biopsy	EF062987[1]	1	KJ424273
					1	120	biopsy	EU833097[4]		

References Cited

1. Lee T, Burch JB, Jung Y, Coote T, Pearce-Kelly P, Ó Foighil D: **Tahitian tree snail mitochondrial clades survived recent mass-extirpation.** *Curr Biol* 2007, **17:**R502-R503.

2. Lee T, Burch JB, Coote T, Fontaine B, Gargominy O, Pearce-Kelly P, Ó Foighil D: **Prehistoric inter-archipelago trading of Polynesian tree snails leaves a conservation legacy.** *Proc R Soc Lond B* 2007, **272:**2907-2914.

3. Lee T, Meyer J-Y, Burch JB, Pearce-Kelly P, Ó Foighil D: **Not completely lost: two partulid tree snail species persist on the highest peak of Raiatea, French Polynesia.** *Oryx* 2008, **42:**615-619.

4. Lee T, Burch JB, Coote T, Pearce-Kelly P, Hickman C, Meyer J-Y, Ó Foighil D: **Moorean tree snail survival revisited: a multi-island genealogical perspective.** *BMC Evolutionary Biology* 2009, **9:**204

5. Ó Foighil D, Lee T, Slapcinsky J: **Prehistoric anthropogenic introduction of partulid tree snails in Papua New Guinean archipelagos.** *J Biogeogr*. 2011, **38**:1625-1632.

6. Rundell, RJ, Holland, BS, Cowie RH: **Molecular phylogeny and biogeography of the endemic Hawaiian Succineidae (Gastropoda: Pulmonata).** *Mol Phylogenet Evol* 2004, **31**:246-255.
